# Genome-wide identification, evolutionary and expression analysis of the aspartic protease gene superfamily in grape

**DOI:** 10.1186/1471-2164-14-554

**Published:** 2013-08-15

**Authors:** Rongrong Guo, Xiaozhao Xu, Bassett Carole, Xiaoqin Li, Min Gao, Yi Zheng, Xiping Wang

**Affiliations:** 1 State Key Laboratory of Crop Stress Biology in Arid Areas, College of Horticulture, Northwest A&F University, Yangling, Shaanxi 712100, China; 2 Key Laboratory of Horticultural Plant Biology and Germplasm Innovation in Northwest China, Ministry of Agriculture, Northwest A&F University, Yangling, Shaanxi 712100, China; 3 Agricultural Research Service, Appalachian Fruit Research Station, United States Department of Agriculture, Kearneysville WV 25430, USA

**Keywords:** Synteny analysis, Phylogenetic analysis, Gene expression, Orthologous genes, Grape

## Abstract

**Background:**

Aspartic proteases (APs) are a large family of proteolytic enzymes found in almost all organisms. In plants, they are involved in many biological processes, such as senescence, stress responses, programmed cell death, and reproduction. Prior to the present study, no grape AP gene(s) had been reported, and their research on woody species was very limited.

**Results:**

In this study, a total of 50 AP genes (*VvAP*) were identified in the grape genome, among which 30 contained the complete ASP domain. Synteny analysis within grape indicated that segmental and tandem duplication events contributed to the expansion of the grape AP family. Additional analysis between grape and *Arabidopsis* demonstrated that several grape AP genes were found in the corresponding syntenic blocks of *Arabidopsis*, suggesting that these genes arose before the divergence of grape and *Arabidopsis*. Phylogenetic relationships of the 30 *VvAPs* with the complete ASP domain and their *Arabidopsis* orthologs, as well as their gene and protein features were analyzed and their cellular localization was predicted. Moreover, expression profiles of *VvAP* genes in six different tissues were determined, and their transcript abundance under various stresses and hormone treatments were measured. Twenty-seven *VvAP* genes were expressed in at least one of the six tissues examined; nineteen *VvAPs* responded to at least one abiotic stress, 12 *VvAPs* responded to powdery mildew infection, and most of the *VvAPs* responded to SA and ABA treatments. Furthermore, integrated synteny and phylogenetic analysis identified orthologous AP genes between grape and *Arabidopsis*, providing a unique starting point for investigating the function of grape AP genes.

**Conclusions:**

The genome-wide identification, evolutionary and expression analyses of grape AP genes provide a framework for future analysis of AP genes in defining their roles during stress response. Integrated synteny and phylogenetic analyses provide novel insight into the functions of less well-studied genes using information from their better understood orthologs.

## Background

Aspartic proteinases (APs; EC 3.4.23) are widely distributed among living organisms, being found in plants, yeast, nematodes, parasites, fungi and even viruses. These enzymes have been extensively studied and constitute one of the four superfamilies of proteolytic enzymes [1-3]. APs are usually characterized by the presence of two aspartic acid residues located within the conserved Asp-Thr/Ser-Gly motif responsible for catalytic activity [4]. They are active at acidic pH and are specifically inhibited by active site blockers such as pepstatin A, diazo-acetyl-norleucine methyl ester (DAN), and 1,2-epoxy-3-(p-nitro-phenoxy) propane (EPNP) [5]. APs are synthesized as single-chain preproenzymes which are subsequently converted to mature enzymes that can function as either monomeric or dimeric proteins during activation. According to the MEROPS database (http://www.merops.ac.uk), APs are now grouped into 14 different families on the basis of their amino acid sequence homology, evolutionary relationships, and tertiary structure; these groups in turn are assembled into six different clans [3]. Plant APs are distributed among several different families (A1, A3, A11 and A12 of clan AA, and family A22 of clan AD), but the majority belong to the A1 family [6].

Plant APs are classified as typical APs, nucellin-like APs and atypical APs [7]. Typical plant AP preproteins contain a C-terminal domain of approximately 50–100 amino acids (called the plant specific insert, PSI) which is removed during protein maturation. Neither their sequences nor structures share significant homology with animal or microbial APs; however, the PSI domain is homologous with the precursor of mammalian saposins [8]. The nucellin-like APs encode proteins similar to nucellin found in barley nucellar cells [9]. Atypical APs display intermediate features between the typical and nucellin-like sequences [7].

Plant APs have been implicated in protein processing and/or degradation in different plant organs. They are believed to play a role in plant senescence, stress responses, programmed cell death, and reproduction [6]. In contrast to APs of animal and microbial origin, plant APs are relatively poorly documented with regard to their biochemistry and physiological functions [10]. Furthermore, most of the analyses on plant APs have been performed in model species such as *Arabidopsis*[7,11], with little attention paid to woody species like grape.

Grapevine (*Vitis vinifera* L.) is one of the most important perennial fruit crops worldwide. It has been extensively studied at the physiological and developmental levels and was among the first fruits selected for full genome sequencing [12]. Compared to other perennials, the genome size of *V. vinifera* is relatively small (475 Mb) [12,13], which is similar to rice (*Oryza sativa*, 430 Mb) [14] and black cottonwood poplar (*Populus trichocarpa*, 465 Mb) [15]. In addition, the grapevine genome has not undergone a recent whole genome duplication (WGD), thus enabling the discovery of ancestral traits and genetic divergence occurring during the course of flowering plant evolution [12]. The release of grape genome data allows us for the first time to carry out the genome-wide identification and analysis of AP gene families in a woody species. Here we systematically identified 50 AP genes including 30 *VvAPs* that contain a complete ASP domain in the grape genome. Phylogenetic and synteny analyses revealed segmental and tandem duplication events that have contributed to the grape AP evolution. We further analyzed protein structures and exon/intron junctions of *VvAPs*. In addition, we determined the expression profiles of grape AP genes in six different tissues, and measured their transcript abundance in response to different phytohormone treatments and under various abiotic and biotic stresses. The results obtained from our study provide a foundation for evolutionary and functional characterization of AP gene families in grape and other plant species.

## Methods

### Identification and annotation of grape AP genes

Grape AP genes were identified by searching grape proteins obtained from the Grape Genome Database (12×; http://www.genoscope.cns.fr) using the Hidden Markov Model (HMM) profile of ASP domain (PF00026) downloaded from the Pfam database (http://pfam.sanger.ac.uk/). The ASP domain in each identified AP gene was then manually checked for its completeness.

### Determination of chromosomal localization and synteny analysis

The *VvAP* genes were positioned on grape pseudomolecules available at the Grape Genome Database (12 X). Tandemly duplicated AP genes in the grape genome were defined as adjacent to homologous AP genes on the grape chromosomes or within a sequence distance of 50 kb [16], with no more than one intervening gene [17]. For synteny analysis, synteny blocks within the grape genome and between grape and *Arabidopsis* genomes were downloaded from the Plant Genome Duplication Database and those containing grape AP genes were identified.

### Sequence alignments and phylogenetic analysis

A total of 20 APs that lack a complete ASP domain, including *VvAP1*, *VvAP2*, *VvAP5, VvAP7*, *VvAP11*, *VvAP14*, *VvAP15*, *VvAP19*, *VvAP20*, *VvAP22 VvAP23*, *VvAP26*,*VvAP31*, *VvAP37*, *VvAP38*, *VvAP43*, *VvAP47*, *VvAP49* and *VvAP50*, were excluded from the phylogenetic analysis and further study. The remaining 30 *VvAPs* and *AtAPs* were aligned using ClustalX [18]. To compare and define subgroups, we integrated conserved ASP domain sequences of nucellins from barley (GenBank accession no. U87148) [9] into this dataset. The following *Arabidopsis* genes were selected for predicting the function of their orthologous counterparts in grape: *CDR1* (NP_198319, which plays a crucial role in activating resistance of *Arabidopsis* against microbial pathogens) [11]; *PCS1* (NP_195839, the PROMOTION of CELL SURVIVAL 1 gene, which encodes an aspartic protease with an important role in determining the fate of both male and female gametophytes and excessive cell death of developing embryos) [19]; and *ASPG1* (NP_188478, the ASPARTIC PROTEASE IN GUARD CELL 1 gene whose over expression conferred drought avoidance via ABA-dependent signalling in *Arabidopsis*) [20]. Phylogenetic trees were constructed with the MEGA 5.0 software using the neighbor-joining (NJ) method, and the bootstrap test was replicated 1000 times [21].

### Exon/intron structure analysis of *VvAP* genes

The 30 *VvAP* genes with the complete ASP domain were used in this study. The Pfam domain and signal peptide were predicted using SMART (http://smart.embl-heidelberg.de/smart/set_mode.cgi?NORMAL=1) [22]. The diagram of protein structures was constructed with the DOG 1.0 software (http://dog.biocuckoo.org/) [23]. The exon/intron structures of the grape AP genes were determined from alignments of their coding sequences with corresponding genomic sequences using the est2genome program [24]. The diagram of exon/intron structures was obtained using the online Gene Structure Display Server (GSDS: http://gsds.cbi.pku.edu.ch) [25], which exhibited both exon position and gene length.

### Plant materials

Grape tissues, including young roots, stems, leaves, and tendrils, flowers at the fully opening stage, and fruits at 33 days post anthesis were harvested from two year-old ‘Kyoho’ (*V. labrusca* × *V. vinifera*) seedlings grown in the field. ‘Kyoho’ was also used for high salt, drought stress, and exogenous hormone treatments. Chinese wild *Vitis quinquangularis* ‘Shang-24’ was used for powdery mildew inoculation. Both grape species are maintained in the grape germplasm resource orchard of Northwest A&F University, Yangling, China (34°20’N, 108°24’E).

### Abiotic, hormone and biotic stress treatment

For abiotic stress, two year-old ‘Kyoho’ grape seedlings planted in pots were irrigated with 2 dm^3^ 250 mM NaCl [26,27]. After treatments for 1 h, 3 h, 6 h, 12 h, 24 h and 48 h, the fully unfolded young leaves were collected. Drought stress was carried out by withholding water from ‘Kyoho’ seedlings with some modification [28,29]. Briefly, young leaves of the seedlings were harvested at 24 h, 48 h, 72 h, 96 h, 120 h, 144 h and 168 h post treatment. Subsequently, the stressed plants were rewatered to soil saturation and leaves were collected at 48 h after rewatering. For salt and drought stress, plants watered every three days were used as control.

Hormone treatments were conducted by spraying young leaves with 100 μM SA [30,31] or 100 μM ABA [26,32] followed by sampling at 0.5 h, 1 h, 3 h, 6 h, 12 h, 24 h and 48 h post-treatment. Leaves sprayed with sterile distilled water at the same time points were collected as the control.

Pathogen treatment was carried out by inoculating the young leaves of ‘Shang-24’ with powdery mildew as previously described with minor modifications [33]. Prior to inoculation, leaves were sprayed with sterile water, and leaves were harvested at 6 h, 12 h, 24 h, 48 h, 72 h, 96 h and 120 h post-inoculation (Hpi). Control plants were simply sprayed with sterile water and not inoculated.

At each time point of each treatment, nine leaves from three separate plants were combined to form one sample. These leaves were immediately frozen in liquid nitrogen and stored at −80°C until use.

Several genes that have been reported to positively respond to abiotic or biotic stress were used to confirm the efficacy of the stress treatments; these included *RD22* and *CEF4* (induced by drought, salt and/or ABA treatments) [34-36], and *PR1* and *EDS1* (enhanced by SA treatment and powdery mildew inoculation) [37-40] (Additional file 1).

### Semi-quantitative RT-PCR and real-time PCR analysis

Total RNA was extracted according to Zhang et al. [41], and then treated with 10 units of RNase-free DNase I (TaKaRa Bio Inc., Dalian, China) to remove genomic DNA contamination. For each sample, 1 μg of total RNA was used to synthesize first-strand cDNA using SuperScriptII reverse transcriptase (Invitrogen). For the following experiments, the reverse transcription products were diluted to six times. The concentration of the cDNAs was adjusted using the grape *Actin1* gene (GeneBank Accession number AY680701) with the primers F (5′-GAT TCT GGT GAT GGT GTG AGT-3′) and R (5′-GAC AAT TTC CCG TTC AGC AGT-3′) and the grape *EF1-α* gene (GeneBank Accession number EC931777) with the primers F (5′-AGG AGG CAG CCA ACT TCA CC-3′) and R (5′-CAA ACC CTG CAT CAC CAT TC-3′). Gene-specific primers were designed for the 30 grape AP genes with the complete ASP domain (Additional file 2). For semi-quantitative reverse transcription-PCR (RT-PCR), a 20 μl reaction volume that included 1 μl of cDNA template, 1.6 μl of gene-specific primers (1.0 μM), 9.8 μl PCR Master Mix (Tiangen Biotech Co. Ltd., Beijing, China) and 7.6 μl sterile distilled water was used. The PCR parameters were 95°C for 3 min; followed by 25–35 cycles of 95°C for 30s, 58°C for 30s, 72°C for 25 s, and a final step at 72°C for 2 min. Each PCR was replicated three times. The results of semi-quantitative RT-PCR were quantified using the Gene Tools software, and the log-transformed values of the relative transcript abundance of *VvAP* genes under abiotic, hormone and biotic stress treatment compared to the control were used for hierarchical cluster analysis with Genesis software.

Quantitative real-time PCR analysis was conducted with an IQ5 real-time PCR instrument (Bio-Rad, Hercules, CA, USA). Each reaction was carried out in triplicate with a reaction volume of 20 μl containing 1.6 μl of gene-specific primers (1.0 μM), 1.0 μl of cDNA, 10 μl of SYBR green (TaKaRa Bio Inc.), and 7.4 μl sterile distilled water. The PCR parameters were 95°C for 30s, followed by 40 cycles of 95°C for 5 s and 60°C for 30s. Relative expression levels were analyzed using the IQ5 software and the normalized-expression method.

### Database searches of the expression patterns of *AtAP* genes

The expression patterns of *AtAP* genes under powdery mildew infection and SA treatment were obtained from The Gene Expression Atlas of EMBL-EBI (http://www.ebi.ac.uk/gxa/) [42]. The expression patterns of these genes under salt and drought stresses and ABA treatment were obtained from Weigel World database (http://jsp.weigelworld.org/expviz/expviz.jsp) [43].

### Targeting signals prediction of the grape AP genes

The targeting signals of grape AP genes were predicted with PSORT (http://psort.hgc.jp/form.html) [44].

## Results

### Genome-wide identification of AP genes in the *V. vinifera* genome

**Figure 1:**
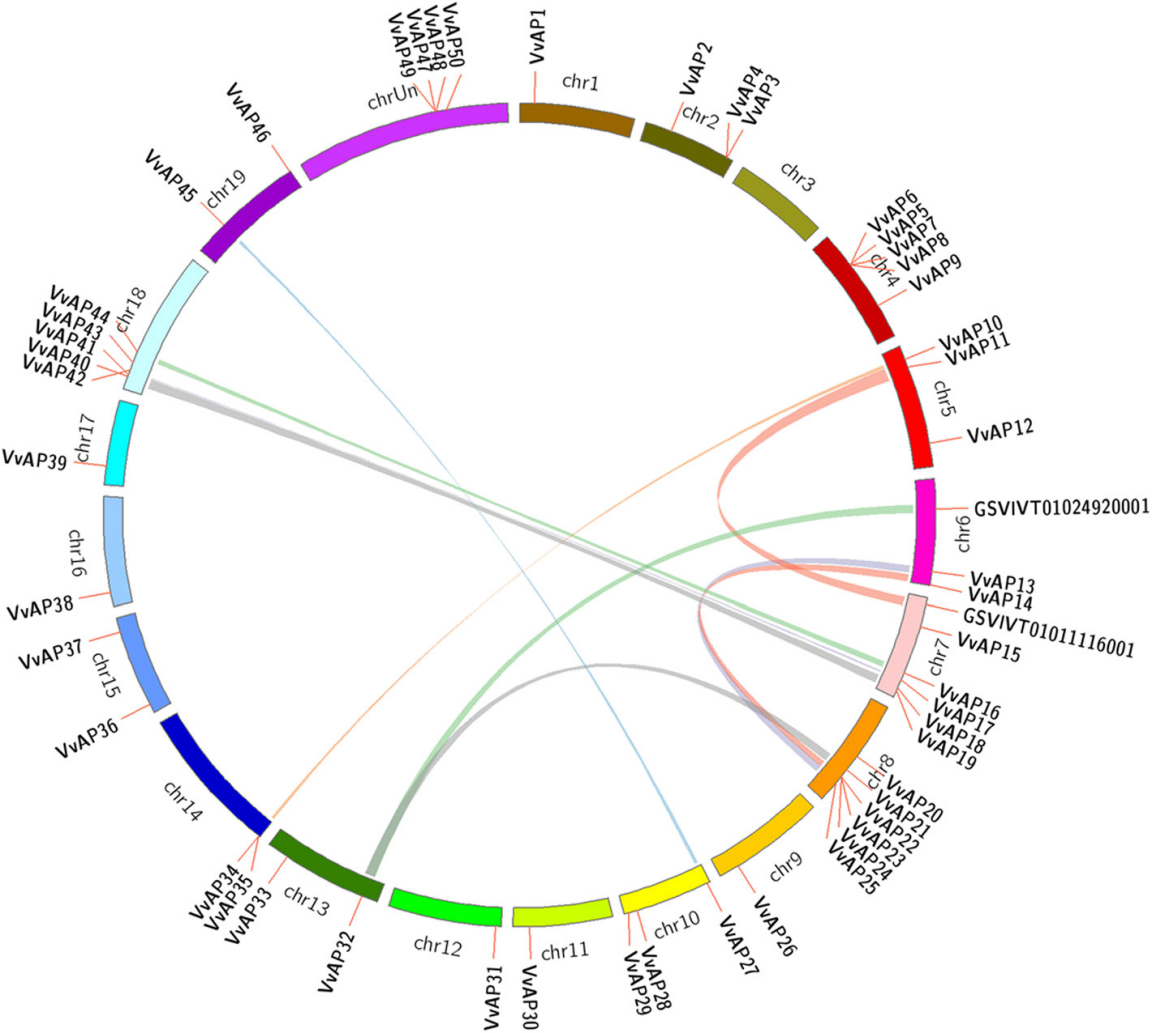
**Distribution and synteny analysis of AP genes on grape chromosomes.** AP genes are indicated by vertical orange lines. Colored bars denote syntenic regions of the grape AP genome.

A total of 50 genes in the grape genome were identified as possibly AP genes (Table 1). Among them, 30 have the two complete Asp-Thr/ Ser-Gly motifs, while the others have either a single motif or an incomplete ASP domain. A total of 46 AP genes could be mapped to specific chromosomes and were named from *VvAP1* to *VvAP46* based on their order on the chromosomes (Figure 1, chromosomes 1–19). Four AP genes (GSVIVT01006876001, GSVIVT01006894001, GSVIVT01006895001 and GSVIVT01007227001) that could not be conclusively mapped to any chromosomes were named *VvAP47-VvAP50*, respectively. Furthermore, additional AP genes could also exist in the grape genome, awaiting identification by improved annotation methodology.

**Table 1:** AP genes in grape

**Group**	**Gene**	**Gene locus ID**	**Accession no.**	**Chromosome**	**Start**	**End**	**Predicted gene length(bp)**	**Predicted ORF length(bp)**
	VvAP1	GSVIVT01011932001	CBI27051.3	chr1	2921237	2927383	6147	771
	VvAP2	GSVIVT01001318001	CBI31923.3	chr2	5263175	5284039	20865	2346
C	VvAP3	GSVIVT01036932001	CBI29231.3	chr2	17430558	17432766	2209	927
C	VvAP4	GSVIVT01036930001	CBI29230.3	chr2	17439361	17448268	8908	1629
	VvAP5	GSVIVT01036013001	CBI21175.3	chr4	7189959	7192597	2639	1161
	VvAP6	GSVIVT01036015001	CBI21177.3	chr4	7244098	7247313	3216	1131
	VvAP7	GSVIVT01036017001	CBI21178.3	chr4	7263244	7279815	16572	1119
C	VvAP8	GSVIVT01036018001	XM_002268722	chr4	7315581	7317297	1717	1383
C	VvAP9	GSVIVT01019071001	XM_002277022	chr4	17094880	17107573	12694	1443
A1	VvAP10	GSVIVT01017701001	CBI26025.3	chr5	2775453	2778471	3019	1503
	VvAP11	GSVIVT01017894001	CBI26188.3	chr5	4453167	4455917	2751	1203
C	VvAP12	GSVIVT01031690001	CBI39464.3	chr5	20228612	20244419	15808	1434
C	VvAP13	GSVIVT01031432001	XM_002272085	chr6	18652672	18658561	5890	1455
	VvAP14	GSVIVT01036053001	CBI28265.3	chr6	21340808	21342747	1939	1341
	VvAP15	GSVIVT01028308001	CBI37099.3	chr7	5960288	5965597	5309	900
C	VvAP16	GSVIVT01000170001	CBI33735.3	chr7	15840160	15846953	6793	1902
C	VvAP17	GSVIVT01022186001	CBI21469.3	chr7	17268969	17274776	5807	1593
B	VvAP18	GSVIVT01022355001	XM_002273952	chr7	18933005	18936614	3610	1281
	VvAP19	GSVIVT01022392001	CBI21639.3	chr7	19580451	19581940	1490	837
	VvAP20	GSVIVT01025811001	CBI32837.3	chr8	11598470	11608650	10181	1368
C	VvAP21	GSVIVT01034136001	CBI30526.3	chr8	14910379	14911704	1326	1140
	VvAP22	GSVIVT01033940001	CBI30375.3	chr8	16541310	16569287	27978	771
	VvAP23	GSVIVT01033937001	CBI30373.3	chr8	16581910	16588393	6484	657
C	VvAP24	GSVIVT01033935001	CBI30372.3	chr8	16595281	16597123	1843	1338
C	VvAP25	GSVIVT01033723001	XM_002263093	chr8	18335899	18342508	6610	1509
	VvAP26	GSVIVT01016100001	CBI25263.3	chr9	18973023	18975323	2301	1080
A1	VvAP27	GSVIVT01012684001	XM_003632893	chr10	634034	638859	4826	1290
C	VvAP28	GSVIVT01026281001	CBI29076.3	chr10	15521671	15522975	1305	1305
C	VvAP29	GSVIVT01034372001	CBI35367.3	chr10	17284723	17286494	1772	1566
C	VvAP30	GSVIVT01010876001	XM_002265511	chr11	16540456	16541682	1226	1227
	VvAP31	GSVIVT01020842001	CBI22091.3	chr12	1179799	1181100	1301	1083
C	VvAP32	GSVIVT01016510001	CBI31649.3	chr13	3269121	3294047	24927	2286
C	VvAP33	GSVIVT01036671001	CBI35120.3	chr13	20059866	20062297	2431	963
A1	VvAP34	GSVIVT01031329001	XM_002276327	chr14	327811	331186	3376	1509
A1	VvAP35	GSVIVT01031327001	CBI39668.3	chr14	337889	340868	2980	1293
C	VvAP36	GSVIVT01019297001	CBI39998.3	chr15	1765842	1766963	1122	1005
	VvAP37	GSVIVT01027158001	CBI40559.3	chr15	17367842	17369993	2152	1311
	VvAP38	GSVIVT01024459001	CBI26572.3	chr16	2849198	2849563	366	366
B	VvAP39	GSVIVT01008267001	CBI15437.3	chr17	3799065	3805978	6913	1422
B	VvAP40	GSVIVT01008844001	CBI18999.3	chr18	2734587	2737586	3000	1173
C	VvAP41	GSVIVT01008883001	CBI19032.3	chr18	3102029	3103369	1341	1149
C	VvAP42	GSVIVT01008978001	CBI19115.3	chr18	3994150	4000643	6494	1566
	VvAP43	GSVIVT01009155001	CBI19249.3	chr18	5617213	5618770	1558	576
C	VvAP44	GSVIVT01009385001	XM_002283222	chr18	7918070	7925298	7229	1980
A1	VvAP45	GSVIVT01037685001	XM_002279013	chr19	6870994	6878047	7054	1545
C	VvAP46	GSVIVT01036694001	CBI24128.3	chr19	23803974	23806961	2988	1137
	VvAP47	GSVIVT01006876001	CBI29432.3	chrUn	28781234	28782604	1371	1011
C	VvAP48	GSVIVT01006894001	XM_002269844	chrUn	28931224	28934832	3609	1557
	VvAP49	GSVIVT01006895001	XM_003635291	chrUn	28936408	28937470	1063	450
	VvAP50	GSVIVT01007227001	CBI25840.3	chrUn	30964385	30966631	2247	906

### Expansion patterns of AP genes in grape

Segmental and tandem duplications are the main mechanisms leading to gene family expansions [45]. Thirty six AP genes involved in tandem duplications had been reported in rice [46]. In the present study, we also identified 11 tandemly duplicated AP genes (*VvAP3/VvAP4, VvAP5-VvAP8, VvAP22-VvAP24,* and *VvAP34/VvAP35* located on grape chromosomes 2, 4, 8 and 14, respectively) (Figure 1). Furthermore, we examined the segmentally duplicated blocks within the grape genome and found that there were 9 pairs of grape AP genes associated with segmental duplications (Figure 1), *VvAP10/VvAP34, VvAP16/VvAP44, VvAP40/VvAP18, VvAP42/VvAP17, VvAP27/VvAP45, VvAP32/VvAP21, VvAP13/VvAP25, VvAP22/VvAP14*, including two tandemly duplicated genes (*VvAP22* and *VvAP34*) (Additional file 3). In summary, half of the *VvAP* gene family members were associated with either segmental or tandem duplication events.

### Evolutionary relationships of AP genes between grape and *Arabidopsis*

To further explore the origin and evolutionary process of grape AP genes, we analyzed the comparative synteny map between grape and *Arabidopsis* genomes. *Arabidopsis* is among the most important model plant species, particularly with regard to AP genes since the functions of some of them have been well characterized. Thus through comparative genomics we can determine the origin and diversification of grape APs based on their *Arabidopsis* homologs.

**Figure 2:**
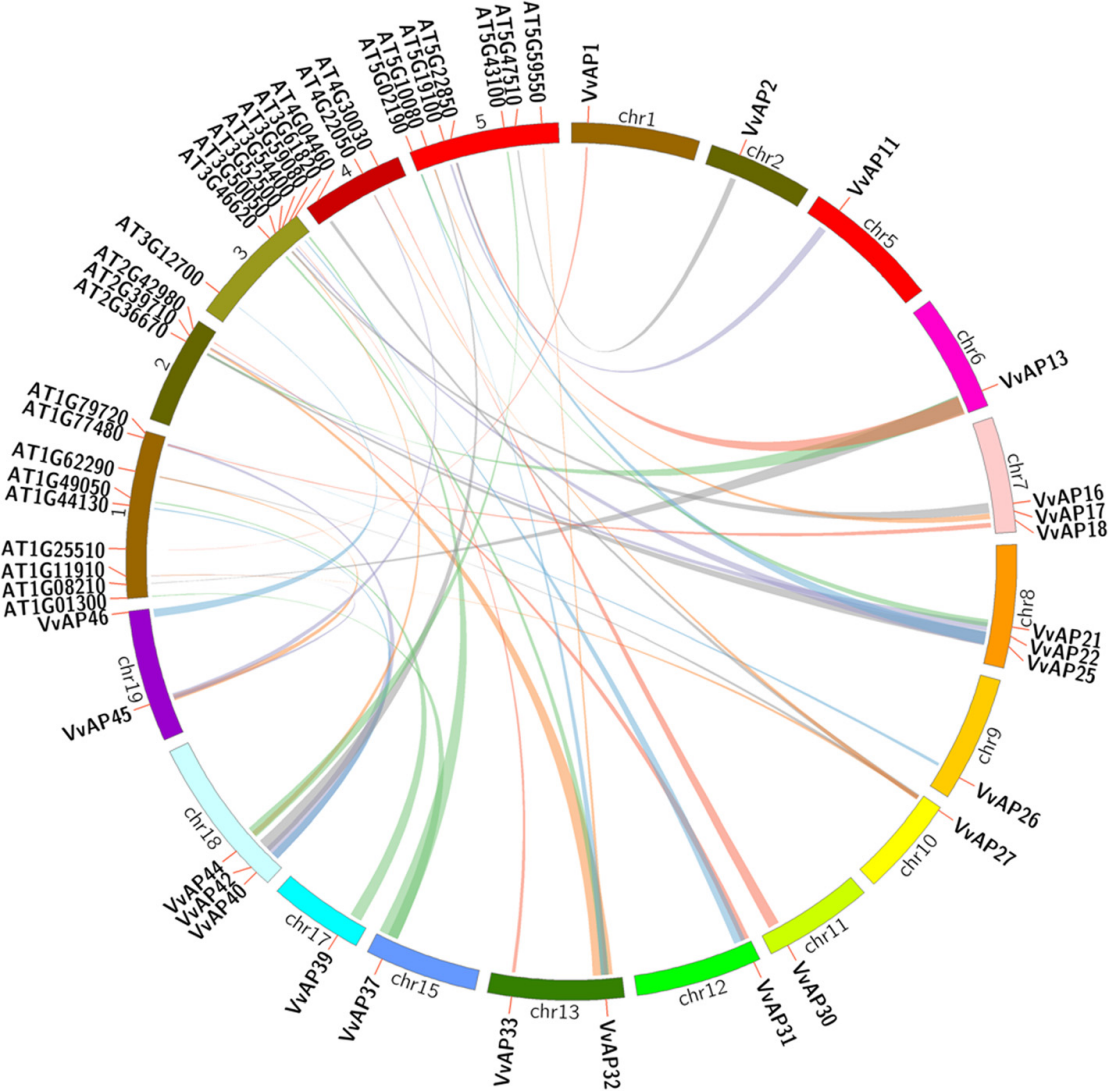
**Synteny analysis of AP genes between grape and *Arabidopsis*.** Grape and *Arabidopsis* AP genes are indicated by vertical orange lines. Colored bars denote syntenic regions between grape and *Arabidopsis* AP chromosomes.

Large-scale syntenies containing 23 AP genes in grape and 25 in *Arabidopsis* were identified (Figure 2). In addition, four genes in the *Arabidopsis* genome that were not annotated as AP genes were found to share synteny with grape AP genes (Additional file 4). Regarding the single grape-to-*Arabidopsis* AP gene correspondences, the syntenies were unambiguous and included the following orthologous pairs: *VvAP1-At1g25510*, *VvAP2-At5g47510*, *VvAP11-At5g19100*, *VvAP22-At3g52500*, *VvAP26-At1g79720*, *VvAP30-At4g30030*, *VvAP33-At3g54400*, *VvAP39-At1g49050*, *VvAP46-At3g12700*, indicating these genes should have been in the genome of the last common ancestor of grape and *Arabidopsis*. Among these, neither *At5g47510* nor *At5g19100* belonged to the *Arabidopsis* AP gene family; instead *At5g47510* has a SEC14 (domain in homologues of a S. cerevisiae phosphatidylinositol transfer protein) and a CRAL-TRIO-N (a protein structural domain that binds small lipophilic molecules) domain and *At5g19100* has a PDB domain 1T6G|B, both of which were also included in *VvAP2* and *VvAP11*, respectively. More challenging for syntenic interpretation were cases where grape segmental duplications corresponded to a single *Arabidopsis* gene or where a single grape gene corresponded to multiple *Arabidopsis* genes. The first situation included *VvAP13/VvAP25-At5g22850*, *VvAP13/VvAP25-At2g36670*, *VvAP16/VvAP44-At3g50050*, *VvAP17/VvAP42-At5g10080*, *VvAP18/VvAP40-At1g77480*, *VvAP21/VvAP32-At2g39710*, *VvAP21/VvAP32-At5g02190*, *VvAP27/VvAP45-At1g11910*, *VvAP27/VvAP45-At1g62290*, *VvAP27/VvAP45-At4g22050*; whereas the second included *VvAP13-At1g08210/At2g36670/At5g22850*, *VvAP21-At2g39710/At5g02190*, *VvAP25-At2g36670/At5g22850*, *VvAP27-At1g11910/At1g62290/At4g04460/At4g22050*, *VvAP31-At2g42980/At3g59080*, *VvAP32-At2g39710/At3g46620/At5g59550/At5g02190*, *VvAP37-At1g01300/At3g61820*, *VvAP40-At1g77480/At1g44130*, *VvAP44-At3g50050/At5g43100*, *VvAP45-At1g11910/At1g62290/At4g22050*. Among these, two of the four orthologs of *VvAP32* in *Arabidopsis* (*At3g46620* and *At5g59550*) were not AP genes; however, both of them had a RING and DUF1117 domain (data not shown), which could also be detected in *VvAP32*, implying that *VvAP32* may have undergone multiple significant chromosomal rearrangement and fusions. Finally, a third case was identified where two duplicated grape genes corresponded to multiple *Arabidopsis* genes. These were *VvAP13/VvAP25-At2g36670/At5g22850*, *VvAP21/VvAP32-At2g39710/At5g02190*, and *VvAP27/VvAP45-At1g11910/At1g62290/At4g22050*.

### Phylogenetic analysis of AP genes from grape and *Arabidopsis*

**Figure 3:**
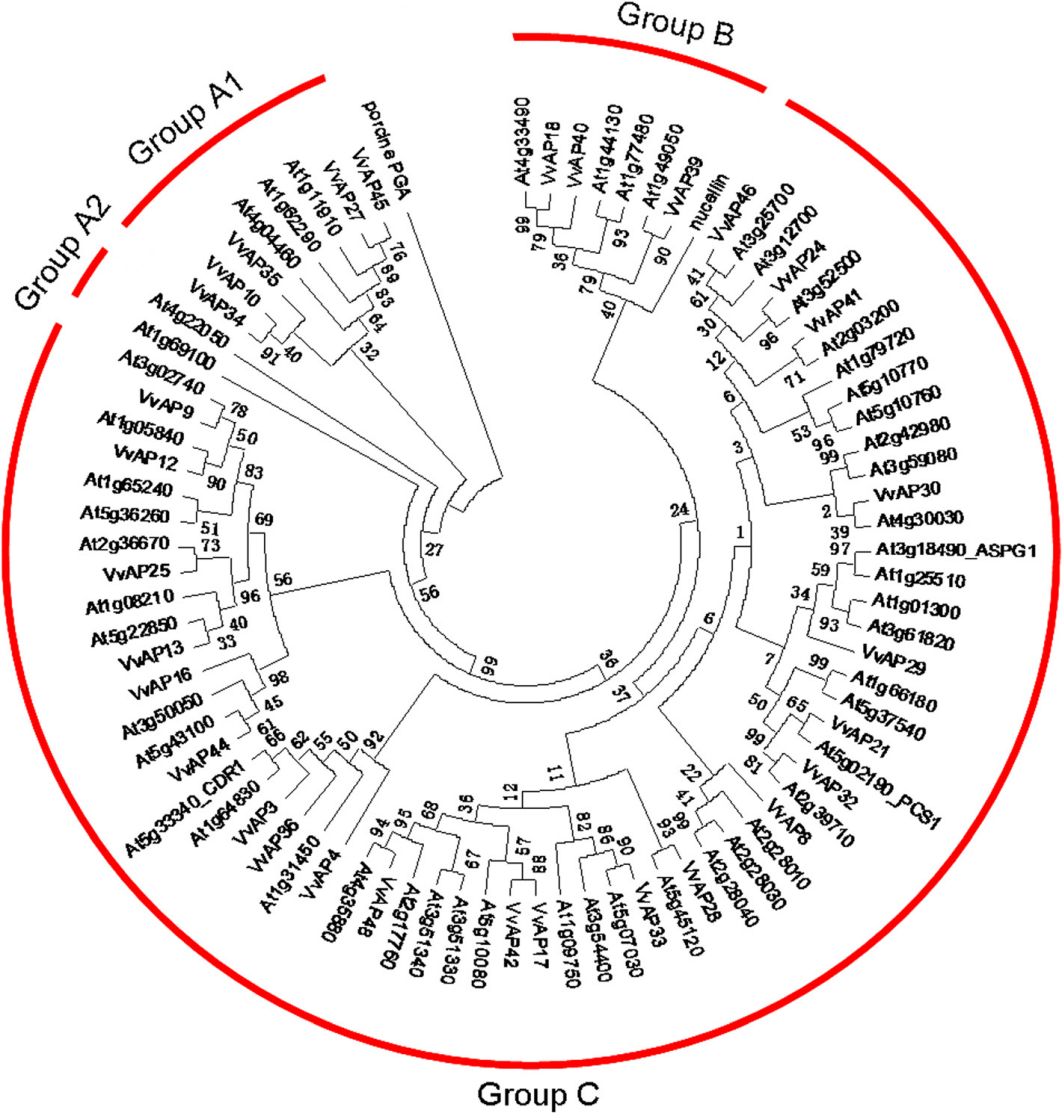
**Phylogenetic analysis of grape and *Arabidopsis *APs.** The conserved ASP domains of all *VvAP* and *AtAP* proteins were aligned with Clustal X 2.0.12, and the phylogenetic tree was constructed using the neighbor-joining method in MEGA 5.0. Porcine pepsin A was used as the outgroup. *VvAPs* that had incomplete conserved ASP domains were not included in the phylogenetic tree analysis.

Among the 50 grape AP genes, 20 have an incomplete conservative ASP domain, and some of them can be mapped to synteny blocks between grape and *Arabidopsis* AP genes (*VvAP1*, *VvAP22*, *VvAP26*, *VvAP31* and *VvAP37*). As a result, we consider them to be members of the grape AP gene family. But since the main focus of this study was to investigate AP members bearing complete ASP domain, these 20 AP genes were therefore not used to construct the phylogenetic tree. The phylogenetic tree was constructed using the conserved ASP domain sequences of the remaining 30 grape AP genes and the *AtAPs*, as well as one family of reference genes (nucellins) (Figure 3). Three categories (A, B and C) were resolved, similar to those described in *Arabidopsis* and rice [7,46]. Category A, which has been divided into A1 and A2 subgroups, containing five *VvAPs* and three *AtAPs* in A1, represented typical aspartic proteases. Two additional *AtAPs* (*At4g22050* and *At1g69100*) were included in subgroup A2; these genes do not have the PSI, but do possess other sequence elements that indicate they are more closely related to typical aspartic proteases than to any other sequences found in the *Arabidopsis* genome. Category B with three *VvAPs* and four *AtAPs* consisted of nucellin-like APs. Category C, composed of atypical aspartic proteases, was the largest group with 22 *VvAP* members and 43 *AtAP* members. Some researchers have divided category C further into five subgroups [7,46], but the criterion for classification was non-uniform, so in this study, the genes in category C were not classified further into subgroups. The rooted phylogenetic tree of ASP domains was also used to identify putative orthologs in *Arabidopsis* and grape (Additional file 5).

### Sequence and structure analysis of grape AP genes

**Figure 4:**
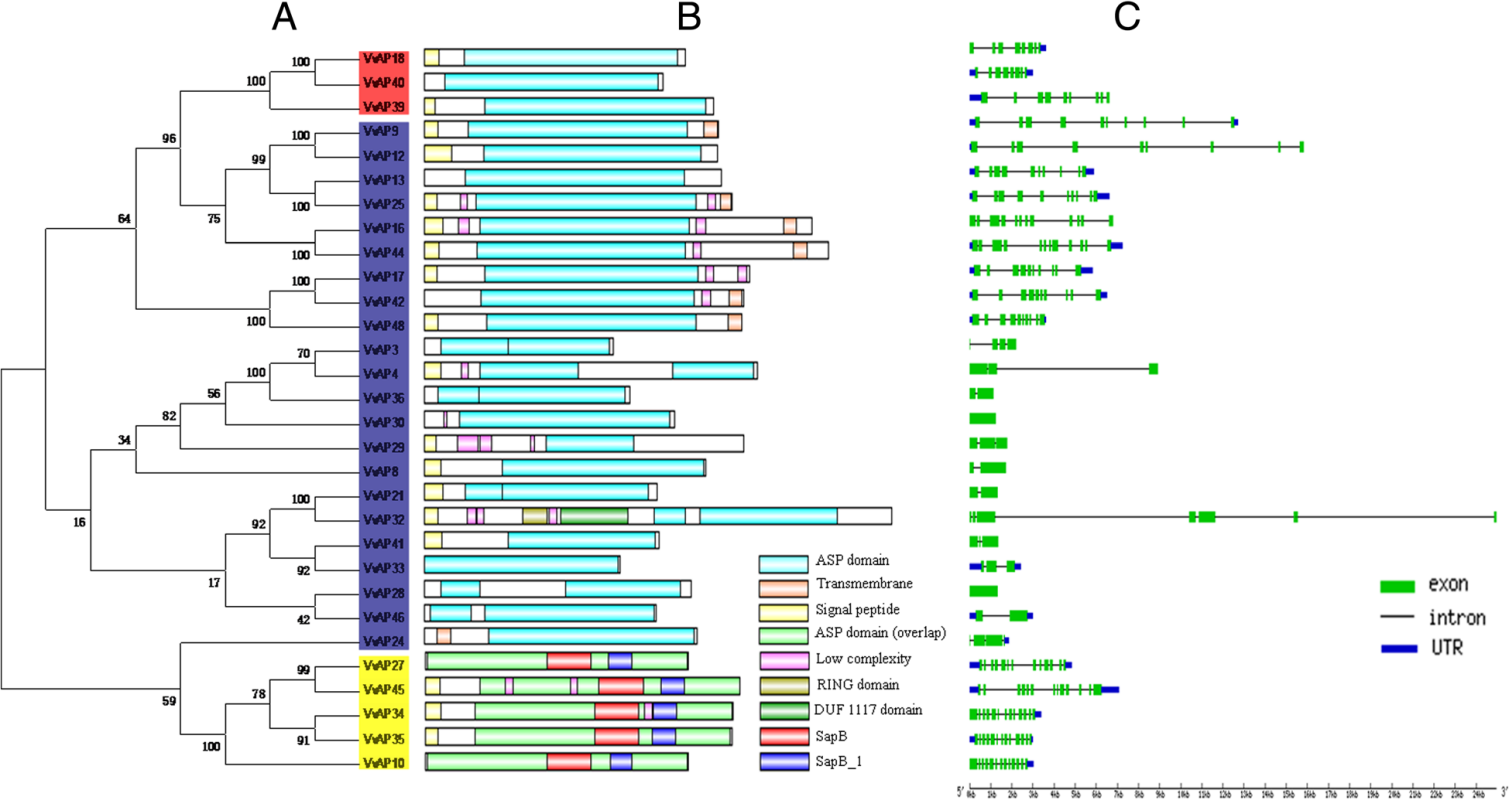
**The AP genes in grape. A**. Phylogenetic analysis of grape AP proteins. Numbers above or below branches of the tree indicate bootstrap values. **B**. The distribution of conserved domains and signal peptide in grape AP proteins. The relative positions of each domain within each protein are shown in color. **C**. Exon/intron structures of grape AP genes.

Phylogenetic analysis was also carried out using the conserved amino acid sequences of the 30 grape AP gene family members identified here (Figure 4A). The topology was similar to that constructed with AP sequences from grape and *Arabidopsis* (Figure 3) and, likewise, AP proteins from the same families within grape clustered together. One exception was the protein *VvAP24*, which had fallen into group C in the two-species analysis, while in the grape analysis it clustered together with the members of group A, being the most divergent member of the AP family.

To provide further confirmation of the evolutionary relationships among the grape AP genes, we determined the distribution of their conserved domains (Figure 4B). By consensus, a *VvAP* protein has the following basic structure: a signal peptide, a propeptide, and an ASP domain with two active sites [47]. All of the 50 *VvAP* proteins identified were predicted to contain at least one ASP domain, but 20 of them had just one active site. These we excluded from the phylogenetic analyses between and within species. Sixty percent of the remaining *VvAP* proteins (18 sequences) had the basic structure of a signal peptide and two active sites. In addition 13 *VvAP* proteins had at least one transmembrane domain or one low complexity domain. Five *VvAPs* were identified as typical *VvAP* proteins, and all of them had a SapB domain and a SapB_1 domain located in the PSI sequence. One atypical protein, *VvAP32*, had a RING domain and a DUF1117 domain.

The divergence of exon/intron structure often plays a key role in the evolution of gene families. Therefore, the exon/intron structures of the grape APs were examined (Figure 4C) to gain further insight into their possible structural evolution. Our results indicated that there was a strong correlation between the phylogeny and exon/intron structure, meaning that genes clustering together generally possessed similar structures. In summary, *VvAP* genes in category A had 13 exons, those in category B ranged from eight to nine. On the other hand, the number of exons in the *VvAP* category C genes varied considerably, with nine of the 22 category C genes ranging from nine to 12 exons, while the others had only one to four exons. *VvAP32* was an exception, having two additional domains and seven exons, suggesting that it may have acquired the two additional domains during evolution.

### Expression profiles of grape AP genes in different tissues

**Figure 5:**
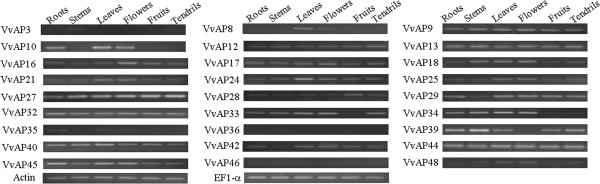
Expression profiles of 27 grape AP genes in various tissues as determined by semi-quantitative RT-PCR analyses.

Semi-quantitative RT-PCR was used to detect the expression patterns of the 30 grape AP genes under normal growth conditions in six different tissues: roots, stems, leaves, flowers, fruits and tendrils. Of these, 27 genes (90%) were expressed in at least one of the six tissues (Figure 5). Expression of the other three genes (*VvAP4*, *VvAP30*, *VvAP41*) was not detected by RT-PCR in any of the tissues tested (data not shown). Twenty-one *VvAP* genes were expressed in all tested tissues but varied in expression levels. For example, *VvAP10* showed high levels of expression in roots, leaves and flowers, but much lower expression in the stems, fruits and tendrils. The other six genes, *VvAP3*, *VvAP8*, *VvAP33*, *VvAP35*, *VvAP36* and *VvAP39*, showed tissue-specific expression patterns. It is worth noting that the transcripts of *VvAP3* and *VvAP36* were neither detected in leaves under normal growth conditions, nor in leaves under stress and hormone treatments (data not shown). Therefore, in addition to *VvAP4*, *VvAP30* and *VvAP41*, these genes were not studied further.

### Expression patterns of grape AP genes under different stress and exogenous hormone treatment conditions

Different approaches have been taken to improve plant stress tolerance, including manipulating and reprogramming the expression of endogenous stress-related genes. Therefore, identification and functional characterization of potential stress-related genes provides fundamental information for future improvement of plant stress tolerance. In the present study, we investigated the response of grape AP genes to various abiotic and biotic stress conditions, as well as hormone treatments.

#### Abiotic stress

**Figure 6:**
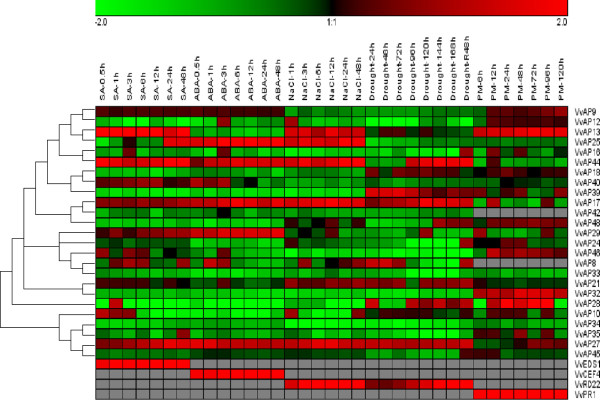
**Hierarchical clustering of grape AP genes.** The results of semi-quantitative RT-PCR were quantified using the Gene Tools software, and the log-transformed values of the relative expression levels of *VvAP* genes under SA, ABA, salt, drought and powdery mildew treatments compared to the controls were used for hierarchical cluster analysis with Genesis software (original results shown in Additional files 6, 7, 8, 9 and 10). The color scale represents relative expression levels with red as increased transcript abundance and green as decreased transcript abundance, gray in the figure represents not detected under the corresponding treatments. Sampling times are indicated at the top of the figure; R48 represents sampling 48 h after recovery from one week (168 h) of drought treatment.

To determine whether *VvAP* genes are responsive to osmotic stresses in grape leaves, semi-quantitative RT-PCR was used to test their transcript abundance under salt and drought stress treatments. As shown in Figure 6 (see also Additional file 6, Additional file 7), 19 genes responded to at least one stress treatment. Three grape AP genes (*VvAP17*, *VvAP27* and *VvAP44*) exhibited enhanced transcript abundance by both treatments, whereas eight (*VvAP8*, *VvAP12*, *VvAP16*, *VvAP32*, *VvAP33*, *VvAP34*, *VvAP35* and *VvAP46*) were down-regulated. In particular, the transcript abundance of *VvAP8, VvAP16, VvAP24, VvAP33, VvAP34* and *VvAP35* decreased gradually with the time under drought stress, but their transcript abundance increased after rewatering, while the other genes exhibited constitutive transcript abundance, or were up- or down-regulated. Interestingly, *VvAP25* was up-regulated in response to salt stress, but down-regulated in response to drought stress. In contrast, *VvAP28* and *VvAP39* transcript abundance decreased when treated with salt, but increased in response to drought. *VvAP13* was up-regulated when exposed to salt stress, while *VvAP10* exhibited decreased transcript abundance; these two genes were not affected by drought treatment. The transcript abundance of *VvAP8, VvAP24* and *VvAP4*5 were decreased by drought stress, but not affected by salt stress. The transcript abundance of the other six genes (*VvAp9*, *VvAP18, VvAP21*, *VvAP29, VvAP40*, *VvAP42*) was not regulated by either osmotic stress treatment. Transcript abundance of three randomly selected AP genes under either treatment were verified by real-time RT-PCR (Additional file 6B and Additional file 7B), indicating that the results of the real-time and semi-quantitative RT-PCR were consistent.

#### Biotic stress

Increasing evidence suggests that AP genes play important roles in response to pathogen infection [11,48]. The stress responses of the 30 grape AP genes were therefore investigated by semi-quantitative RT-PCR to obtain their transcript abundance under powdery mildew infection. *VvAP3*, *VvAP4*, *VvAP30*, *VvAP36* and *VvAP41* whose transcript abundance could not be detected in the leaves of ‘Kyoho’, were also not expressed in the leaves of ‘Shang-24’. Neither *VvAP8* nor *VvAP42* were expressed in the mock leaves or in the inoculated leaves of ‘Shang-24’ (data not shown). Of the remaining 23 genes, half responded to powdery mildew infection, including *VvAP13*, *VvAP28*, *VvAP32* and *VvAP46* which were up-regulated and *VvAP16*, *VvAP17*, *VvAP25*, *VvAP29*, *VvAP33*, *VvAP34*, *VvAP35* and *VvAP44* which were down-regulated (Figure 6 and Additional file 8). The transcript abundance of *VvAP13* in infected leaves peaked at 72 h, whereas *VvAP32* increased slightly at 6 h and remained constant for the duration of the measurement period. *VvAP46* peaked at 12 h post-inoculation. Although the transcript abundance of *VvAP28* in infected leaves was weaker than in mock-inoculated leaves at 6 and 120 h post-infection, its transcript abundance was elevated from 12–96 h post-infection. The efficacy of semi-quantitative RT-PCR was also verified by determining the transcript abundance of three randomly selected AP genes under powdery mildew infection using real-time quantitative RT-PCR (Additional file 8B); both were in agreement.

#### Hormone treatment

The plant hormone, salicylic acid (SA), is a critical factor in plant responses to pathogen infection [49]. Analysis of transcript abundance from ‘Kyoho’ grape leaves sprayed with SA showed that seven of the 25 *VvAPs* (*VvAP13*, *VvAP17*, *VvAP25*, *VvAP27*, *VvAP29*, *VvAP40* and *VvAP44*) were up-regulated by different degrees upon treatment, whereas 11 *VvAP* genes (*VvAP12*, *VvAP16*, *VvAP18*, *VvAP28*, *VvAP32*, *VvAP33*, *VvAP34*, *VvAP35*, *VvAP39*, *VvAP42* and *VvAP48*) were down-regulated. *VvAP10* transcript levels were high at the first three sampling times after SA treatment, but declined thereafter (Figure 6 and Additional file 9).

ABA is known to play a central role in the response of plants to various types of abiotic stress [50]. Analysis of transcript abundance from ‘Kyoho’ leaves treated with exogenous ABA indicated that seven of the 25 *VvAP* genes analyzed exhibited increased expression at different times after treatment (Figure 6 and Additional file 10). For example, the transcript abundance of *VvAP17*, *VvAP25* and *VvAP44* was induced at 3 h after treatment, while that of *VvAP29* was essentially constitutive throughout the experiment. The transcript abundance of *VvAP8* peaked at 1 h after treatment, where as both *VvAP27* and *VvAP40* peaked at 0.5 h post-treatment. Approximately half of the analyzed genes exhibited decreased transcript abundance, whereas the levels of *VvAP9*, *VvAP21*, *VvAP42* and *VvAP45* remained unchanged after treatment.

## Discussion

Members of the plant AP family have been implicated in various physiological and developmental processes, including protein processing and degradation, senescence, stress response, programmed cell death and reproduction. However, virtually nothing is known about this family in woody species. Since grapevine is one of the most important fruit trees worldwide, and various forms of both biotic and abiotic stresses have a serious impact on its production and quality. Further study on stress-related responses in this genus could prove to be a significant asset. Therefore, we have sought to undertake the genome-wide identification of AP genes in grape, and provide clues regarding both their evolutionary histories and expression diversity with respect to stress-related conditions.

### Tandem and segmental duplications contributed to the expansion of the grape AP gene family

Gene duplication, including tandem, segmental and whole genome duplications, has played an important role in the evolution of various organisms [51], and land plants have undergone abundant gene duplication throughout their evolutionary history [52]. Since the grapevine genome has not undergone any recent whole genome duplication events [12], segmental and tandem duplications would be the two main causes of gene family expansions in grape, although there is debate on the exact nature and timing of these events in grape [12,13]. In this study, 26 of 46 grape AP genes which could be precisely located on chromosomes were associated with either tandem or segmental duplication events (Figure 1 and Additional file 3), consistent with findings in rice whereby 51 of 93 AP genes were located in either tandemly or segmentally duplicated regions [46]. Taken together, this suggests that tandem and segmental duplications likely played an important role in the expansion of the AP family in plants. Although the duplicated grape AP genes identified here have a common ancestor, we could not conclude from the work conducted here what the ancestral functions and expression patterns may have been, since gene duplication is considered to provide the raw material for evolution and duplicated genes which, if survive, could undergo substantial changes in their structures and/or regulatory mechanisms allowing them to assume novel roles [53].

### The structural conservative and divergence of grape AP genes

Although several models of the genome evolution have been proposed from comparative genomic analyses of model organisms [54-56], little attention has been paid to the structural evolution of duplicated gene families [57]. In fact, exon/intron diversification of gene family members has played an important role in the evolution of multiple gene families through three main types of mechanism: exon/intron gain/loss, exonization/pseudoexonization, and insertion/deletion [51]. It was obvious that grape AP genes within the same phylogenetic clade (Figure 4A) possessed highly similar exon/intron structures (Figure 4C) and most of the grape AP genes that clustered in the same phylogenetic clade were segmental or tandem duplications (Figure 1 and Figure 4A). Based on the results presented here, it is clear that the expansion of the grape AP family was the result of either segmental or tandem duplication. This is consistent with findings in rice [46], despite the fact that the numbers of exons of the *VvAP* genes in category C (1–4 or 9–12) were different from those of the *OsAP* genes in the same category (less than four).

Exon/intron gain/loss and divergence in exon/intron length which were observed within the coding sequences of several grape AP genes may be the result of chromosomal rearrangement and fusions. A good case was *VvAP32*, which had seven exons, but its paralogous gene, *VvAP21*, had just two exons. Moreover, *VvAP32* contained two other domains (RING and DUF1117) besides the ASP domain, and this might have resulted from a mechanism(s) leading to the additional exons. Divergence either in exon/intron length or exon/intron quantity could potentially lead to the generation of functionally distinct paralogs [51].

### Functional conservation and divergence of tandem and segmental duplicated AP genes in grape

As discussed above, tandem and segmental duplication expanded the grape AP gene family, but it should be noted that only one gene in two of the four tandem duplicated paralogs (*VvAP5/VvAP6/VvAP7/VvAP8*, *VvAP22/VvAP23/VvAP24*) could be included in the phylogenetic tree (Figures 1 and 3), implying that sequences of these genes have been altered to a large extent after gene duplication, and may therefore have lost their original functions or gained new ones.

The grape AP genes involved in the tandem and segmental duplication with complete ASP domains, clustered in the same phylogenetic clade (Figure 4A), and had highly similar exon/intron structures (Figure 4C); however, in different tissues and/or under a variety of stress or hormone treatments, the transcript abundance of the two genes within each pair of paralogs varied from each other (Figures 5 and 6). Although the expression patterns of *VvAP34* and *VvAP35* under each treatment were almost identical, different expression levels could be observed in the same plant organ. The expression of another pair of tandem duplicated paralogs, *VvAP3* and *VvAP4*, were barely detectable under the various treatments, but slight expression of *VvAP3* could be detected in the stems and tendrils of ‘Kyoho’. Regarding the segmental duplication paralogs, almost all of the two AP genes within each pair of paralogs showed different transcript levels under three or four of the five treatments, and exhibited similar transcript abundance under the other one or two treatments. One exception was *VvAP17/VvAP42*, whose transcript levels were totally different under all of the five treatments. These results are similar to findings in rice [46], which showed that not all *OsAP* genes in the same category had similar expression patterns, and *OsAP* genes classified in the same expression pattern might have a distant phylogenetic relationship.

It seems possible that high sequence similarity is not necessarily correlated with similar transcript levels, because proteins with very similar sequences, presumably performing similar biochemical functions, are needed in different tissues and at different periods during growth and development, while at the same time responding to different stresses and hormone treatments. Similar transcript abundance exhibited by different AP genes with dissimilar sequences may perform different biochemical functions, suggesting they may work together in the tissues during growth and development or in response to the same stress or hormone treatment.

It has been reported that duplicated genes rarely diverge with respect to their biochemical function, but instead are limited to alterations in regulatory control [58]. So the different expression profiles between duplicated genes may be caused by varied regulatory network or mutations in the *cis*-regulatory regions [59], or mutations affecting the related regulatory network [60,61]. Epigenetic mechanisms, such as DNA methylation have also been suggested to potentially contribute to the expression divergence of duplicated genes [62,63], where transcriptional silencing has often been associated with DNA methylation in promoter regions [64,65].

A large part of expression divergence is considered to arise through duplication in the course of evolution [45], and functional diversification of the surviving duplicated genes is also considered a major feature of the long-term evolution of polyploids [66]. It has been reported that four types of functional differentiation may follow gene duplication: pseudogenization, conservation of gene function, subfunctionalization and neofunctionalization [67]. Many duplicated genes may be lost from the genome after the duplication events, while neofunctionalization and subfunctionalization contribute to the retention of new genes. The *VvAP* gene family presents an opportunity to study how expression has diverged following gene duplication. Similar transcript abundance between duplicated genes, such as *VvAP34* and *VvAP35*, suggest that the regulatory mechanism of their expression have been conserved; on the other hand, divergence in expression patterns of the duplicated AP genes (neofunctionalization or subfunctionalization) could reflect the acquisition of novel regulatory mechanisms, while silencing of gene expression after duplication leading to nonfunctionalization of the gene implies drastic alteration of the regulatory mechanism.

Besides the possibilities that have been discussed above, the differences in specificity/catalytic properties and cellular localization among/between the duplicated genes could also contribute to the development of different biological functions, leading to the observed expression divergence [6,68,69]. It was found that almost all of the AP genes within each pair of paralogs were located in different parts of the cell, with two exceptions of *VvAP3*/*VvAP4* (both in the nucleus), and *VvAP17*/*VvAP42* (both in the plasma membrane) (Additional file 11). Even if these paralogs shared similar gene structure and cellular localization, the diversity of expression of these genes may be the result of alterations in regulatory sequences occurring shortly after duplication. In addition, alteration of function could also result from the presence or absence of protein-processing enzymes responsible for the activation/deactivation of the enzymes [6].

In summary, diversity in the transcript levels of the duplicated genes may be affected by different and multiple genetic factors depending on the causal duplication mechanism [70], and there maybe cross talk between different treatments or regulatory mechanisms. More research is needed to clarify the specifics of any functional divergence between grape duplicated AP genes, and new factors that may affect transcript divergence and how different factors work together are worth investigation.

### The evolution of AP proteins in grape and *Arabidopsis* and functional prediction of grape AP genes

Genomic comparison is a quick way to transfer knowledge acquired in one taxon for which there is a better understanding of genome structure and function to a less-studied taxon [71]. Thus, the richness of gene functional information known for model plants such as *Arabidopsis* enables one to extrapolate functions of their orthologous genes in other plant taxa. To obtain an overall picture of the grape AP proteins and their relationships with those of *Arabidopsis*, both syntenic and phylogenetic analyses have been performed, and the evolutionary relationship of this gene family within and among the different species has been systematically studied.

There were 23 grape and 25 *Arabidopsis* AP genes, as well as the other four *Arabidopsis* genes that were syntenic orthologous (Figure 2, Additional file 4). Among these, 10 were single grape-to-*Arabidopsis* AP orthologs, indicating these genes come from a common ancestor. The other genes constituted a more complex situation, including ten cases of two grape AP genes that corresponded to one *Arabidopsis* AP gene, 10 cases of one grape AP gene corresponding to multiple *Arabidopsis* AP genes, and three cases of two duplicated grape AP genes that corresponded to multiple *Arabidopsis* AP genes. Certainly, most of the genes included in the complex situation appeared more than once. For example, *VvAP27* correspondence to *At1g11910* and *At1g62290* located on the Chr1 of *Arabidopsis*, as well as *At4g04460* and *At4g022050* located on the Chr4 of *Arabidopsis*, and *VvAP45* correspondence to *At1g11910*, *At1g62290* and *At4022050*, but not to *At4g04460*, so it is impossible to elucidate whether divergence of *VvAP27* and *VvAP45* located in segmental duplications of grape and *At1g11910*, *At1g62290*, *At4022050* and *At4g04460* in *Arabidopsis* occurred prior to or after the divergence of grape and *Arabidopsis* from the last common ancestor. Although 27 grape AP genes could not be mapped into any syntenic blocks, we could not conclude that these genes from grape and *Arabidopsis* did not share a common ancestor. This may be explained by the fact that after the divergence of lineages that led to grape and *Arabidopsis*, their genomes underwent multiple rounds of significant chromosomal rearrangement and fusions, followed by selective gene loss, which can severely obscure the identification of chromosomal syntenies [72]. In such case, it maybe concluded that some of the AP genes in grape and *Arabidopsis* come from a common ancestor, while the others do not. Although the evolutionary histories of grape AP genes could not be established for the period prior to the split between grape and *Arabidopsis* lineages, at least some of the grape genes appeared to share a common ancestor with their *Arabidopsis* AP counterparts.

In order to improve prediction of the functions of specific grape AP genes based on the reported function of their *Arabidopsis* homologs, a phylogenetic tree was constructed, and bootstrap support values (1000 re-sampling) exceeding 50% were used to identify possible orthologous pairs (Figure 3). For example, *VvAP30* and *At4g30030* were clustered together in the phylogenetic tree, but the bootstrap value of their node was no more than 50%. Therefore, *VvAP30* and *At4g30030* were excluded from the orthologous pairs, as were *VvAP13* and *At1g08210/At5g22850*, *VvAP16/VvAP44* and *At3g50050*, *VvAP27* and *At4g04460*, *VvAP17/VvAP42* and *At5g10080*, *VvAP46* and *At3g12700*. There were 11 orthologous pairs in the phylogenetic tree that could not be detected in the syntenic orthologs, and 21 syntenic orthologs that could not be detected or were not clustered together in the phylogenetic tree (Additional file 4, Additional file 5, Additional file 12). Thus, there were ten orthologs including 8 grape AP genes (*VvAP27/VvAP45-At1g11910/At1g62290*, *VvAP39-At1g49050*, *VvAP21-At5g02190*, *VvAP25-At2g36670*, *VvAP32-At2g39710*, *VvAP33-At3g54400* and *VvAP44-At5g43100*) that could be clustered together in the phylogenetic tree and were also contained in the syntenic map (Additional file 5, Additional file 12). Expression patterns of the ten orthologs were more similar than other orthologous pairs that only clustered together in the phylogenetic tree or were syntenic orthologs (Additional file 12). As a result, we can speculate that the functions of the eight grape AP genes are more similar to their *Arabidopsis* homologs than the other grape APs in the phylogenetic tree and syntenic map. Ling et al. [73] has used phylogeny-based methods to identify orthologs between *Arabidopsis* and cucumber, and further analyzed the correlation of roles of orthologous pairs under abiotic stresses. Their results showed that correlative expression profiles in stress-inducible orthologous WRKY genes between cucumber and *Arabidopsis* and orthologous WRKY genes with different evolutionary patterns displayed a low correlation in their expression patterns [73]. Our study combining synteny analysis with a phylogenetic tree provides new insight for investigating the function of grape AP genes by comparing orthologous genes between two plants, in one of which functional roles for the genes have been identified, in this case, between grape and *Arabidopsis*.

### VvAP proteins play important roles in a range of biological processes

It has been reported that plant APs are implicated in a variety of biological processes [6,7]. The study on the rice AP gene family showed that 66 genes were presented in at least one of the developmental stages analyzed [46]. Timotijevic et al. [74] isolated an aspartic proteinase gene *FeAP12* from developing buckwheat seeds, and found the gene was seed-specifically expressed. Moreover, the highest levels of *FeAP12* expression were observed in the early stages of seed development, suggesting a potential role in nucellar degradation [74]. Our RT-PCR results showed that most of the *VvAP* genes exhibited diverse expression levels in all six organs, indicating these APs often participate in plant development. Genes which showed higher expression levels in one organ than in others may play key roles in the development process of the corresponding organ. It was worth noting that the expression of *VvAP36* could only be detected in stems, indicating its potential role in the stem development.

Evidence is accumulating that AP proteins are involved in plant responses to various abiotic and biotic stresses. Cruz et al. [75] reported that in drought-susceptible common bean cultivars subjected to water deficit, the expression of an AP gene was shown to be transcriptionally up-regulated and its activity was significantly increased. In recently published reports, Yao et al. [20] have shown that an *Arabidopsis* gene, *ASPG1* (*aspartic protease in guard cell 1*), may function in drought avoidance through abscisic acid (ABA) signaling in guard cells. An aspartic protease gene, *FeAP9*, whose expression was up-regulated in leaves under different abiotic stresses has also been found in developing organs of buckwheat [69]. In the present study, we showed that 18 *VvAP* genes exhibited differential transcript abundance in response to at least one abiotic stress (Figure 6), indicating that *VvAP* genes may play an important role in protecting grape from abiotic stresses.

Expression of an extracellular AP gene has also been detected in tobacco and tomato leaves and implicated in the degradation of pathogenesis-related (PR) proteins. It has been suggested that APs may play a role in a conserved mechanism for PR-protein turnover, preventing over accumulation and thereby regulating the biological functions of these stress induced proteins [76,77]. These APs were also shown to be constitutively expressed either in healthy or infected leaves, which was consistent with our findings in this study. Studies with potato showed that the expression levels of *StAPs* were associated with the degree of resistance of potato cultivars to *Phytophthora infestans*, and potato aspartic proteinases were components of the plant defense response [78]. Xia et al. [11] have also shown the accumulation of an AP gene, *CDR1* (*Constitutive disease resistance*), in response to pathogen attacks. The *CDR1* gene in rice has also been studied, and the results suggested that *OsCDR1* was implicated in disease resistance signaling [10]. Powdery mildew, caused by the obligate biotrophic fungus, *Uncinula necator*, has a serious impact on grape productivity and fruit quality [79]. As shown in Figure 6, four of the grape AP genes exhibited increased transcript abundance in the infected leaves, indicating these genes may participate in the plant response to powdery mildew infection. It has been reported that the PSI may take part in defensive mechanisms against pathogens and/or as an effector of cell death [6], but none of the grape AP genes in group A1 was up-regulated upon powdery mildew infection. However, we cannot conclude that the *VvAPs* in group A1 have no function in defensive against pathogens, because they may participate in resistance against other pathogens.

Besides the functions of APs in response to abiotic and/or biotic stress, some APs were reported to be involved in PCD (programmed cell death) [80]. In addition, nucellin, an AP belonging to group B subfamily and known to be expressed specifically in nucellar cells during degeneration after ovule fertilization in barely, was suggested to be involved in PCD [9]. So we can speculate that *VvAPs* in group B is also involved in various types of PCD [81].

Differences of the cellular localization of AP genes may result in their different biological functions [6,68,69], and it has been reported that most plant APs were vacuolar enzymes [82-85], or were secreted to the cell wall[86,87]. But there were many aspartic proteinases in *Arabidopsis* and one third of the grape AP genes were predicted to be localized to the chloroplast and chloroplast thylakoid membrane, respectively [7] (Additional file 11). In tobacco, one chloroplast-located AP gene named *CND41* (for 41 kD Chloroplast Nucleoid DNA-binding protein) is involved in degradation of the Rubisco holoprotein during leaf senescence, and the accumulation of *CND41* is negatively correlated with chloroplast transcript levels in tobacco cells [88-90]. The homologs of *CND41* in *Arabidopsis* have also been confirmed to participate in the regulation of Rubisco turnover and senescence [91-93]. In a more recently published report, Paparelli et al. [94] have identified a chloroplast-located AP gene *NANA* whose misexpression or overexpression not only influences photosynthetic carbohydrate metabolism but also plastid and nuclear gene expression [94]. So the localization of these AP genes to the *Arabidopsis* or grape thylakoid membranes raises the possibility that they may fulfill roles as specific processing enzymes in this organelle, or participate in maintenance or degradation of photosystem proteins [7,95].

To get a more complete understanding of the biological functions of the AP gene family, identification of substrates that AP proteins act on and the regulatory network of AP genes participating in response to various pathogens are necessary. The results presented here indicate that the regulatory role of AP proteins under abiotic and biotic stress is complex and more work is needed to understand the regulatory mechanisms.

## Conclusions

In the present study, we identified a total of 50 grape AP genes, 30 of which had complete ASP domains. Synteny analysis within grape demonstrated that segmental and tandem duplications have contributed to the expansion of the grape AP gene family. Comparative synteny analysis between the *V. vinifera* and *Arabidopsis* genomes indicated that some of the grape and *Arabidopsis* AP genes were located in syntenic regions, suggesting that these genes had common ancestors. Separation of the grape AP genes into three groups was mutually supported by their phylogeny, exon/intron structure and the distribution of conserved domains. Finally, we analyzed expression profiles of 30 *VvAP* genes that possess a complete ASP domain under normal growth conditions and in response to various abiotic and biotic stresses and hormone treatments. Some of the genes evaluated were not expressed in leaves of ‘Kyoho’ and/or ‘Shang-24’. The expression information reported here will be useful for further investigation of the function of AP genes under various stress conditions. Although the genome sequence of grape has been reported, functional studies on grape genes still lag behind. We integrated the synteny analysis between grape and *Arabidopsis* with a phylogenetic tree of the two species, and found that there were ten orthologs, including eight grape AP genes clustered together in the phylogenetic tree and also contained in the syntenic map. Although correlation of the expression of orthologous AP genes in grape and *Arabidopsis* was not calculated, the method of combining synteny analysis with the phylogenetic tree may provide a new approach for investigating gene function from their orthologs whose functions have been previously clarified. These studies could increase our understanding of the roles of these genes in grape, but further functional analysis of stress-responsive *VvAPs* is required to confirm their role in stress tolerance.

## Abbreviations

AP: Aspartic protease; CDR1: Constitutive disease resistance gene 1; PCS1: Promotion of cell survival gene 1; ASPG1: Aspartic protease in guard cell 1; WGD: Whole genome duplication; NJ: Neighbor-joining; SA: Salicylic acid; ABA: Abscisic acid; RT-PCR: Reverse transcription PCR; Hpt: Hours post-treatment; R48: 48-hour after rewatering; Hpi: Hours post-inoculation; PR: Pathogenesis-related; PCD: Programmed cell death.

## Competing interests

The authors declare that they have no competing interests.

## Authors’ contributions

XW, RG designed the study. RG and YZ performed data analysis. XX, MG and XL contributed to abiotic and hormone treatments. RG and XL did the RT-PCR and qRT-PCR analysis. XW provided guidance on the whole study. RG and XW wrote and revised the manuscript. CLB assisted with the interpretation of the results and provided editorial support for the manuscript. All authors approved the final manuscript.

## Supplementary Material

Additional file 1 **The primer sequences used for semi-quantitative RT-PCR of the selected positive control genes for salt, drought, powdery mildew, SA and ABA treatments.** The specific primers were obtained from published reports. Click here for file

Additional file 2 **The primer sequences used for semi-quantitative RT-PCR amplification of 30 *VvAP *genes.** The specific primers were designed according to the AP gene sequence by Primer 5.0 software. Click here for file

Additional file 3 **Synteny blocks of AP genes within the grape genome.** The data were downloaded from the Plant Genome Duplication Database, and those containing grape AP genes were identified. Click here for file

Additional file 4 **Synteny blocks of AP genes between grape and *Arabidopsis *genomes.** The data were downloaded from the Plant Genome Duplication Database, and those containing grape AP genes were identified. Click here for file

Additional file 5 **Putative orthologs of grape and *Arabidopsis*.** Grape AP proteins and their putative orthologs in *Arabidopsis* were identified based on phylogenetic studies of ASP domain sequences. Click here for file

Additional file 6 **Expression patterns of grape AP genes under salt treatment condition.** A. Expression patterns of 25 AP genes under salt treatment conditions were determined by semi-quantitative RT-PCR analyses. For each gene, the upper six amplification bands represent amplified products from leaves of ‘Kyoho’ after treatment with 2 dm^3^ 250 mM NaCl; the bands under them represent amplified products from leaves of the control. B. Expression patterns of three randomly selected AP genes were analyzed by real-time RT-PCR. The grape *Actin1* and *EF1-α* genes were used as internal controls to normalize the data. *VvRD22* served as a positive control for salt stress. The error bars were calculated based on three replicates. Click here for file

Additional file 7 **Expression patterns of grape AP genes under drought treatment.** A. Expression patterns of 25 AP genes under drought treatment conditions were determined by semi-quantitative RT-PCR analyses. For each gene, the upper eight amplification bands represent amplified products from leaves of ‘Kyoho’ under drought stress for 24 h, 48 h, 72 h, 96 h, 120 h, 144 h, 168 h after 48 h of recovery (R48; rewatered); the bands under them represent amplified products from leaves of the control. B. Expression patterns of three randomly selected AP genes were detected by real-time PCR. *VvRD22* was used as a positive control. Click here for file

Additional file 8 **Expression patterns of grape AP genes under powdery mildew treatment.** A. Expression patterns of 23 AP genes under PM treatment condition were determined by semi-quantitative RT-PCR analyses. For each gene, the upper seven amplification bands represent amplified products from leaves of ‘Shang-24’ after inocululation with powdery mildew; the bands under them represent amplified products from Mock-inoculated leaves. B. Expression patterns of three randomly selected AP genes were detected by real-time PCR. *VvPR1* was used as a positive control. Click here for file

Additional file 9 **Expression patterns of grape AP genes under SA treatment.** A. Expression patterns of 25 AP genes under SA treatment conditions were determined by semi-quantitative RT-PCR analyses. For each gene, the upper seven amplification bands represent amplified products from leaves of ‘Kyoho’ after treatment with 100 μM SA; the bands under them represent amplified products from control leaves. B. Expression patterns of three randomly selected AP genes were analyzed by real-time PCR. *VvEDS1* was used as a positive control. Click here for file

Additional file 10 **Expression patterns of grape AP genes under ABA treatment.** A. Expression patterns of 25 AP genes under ABA treatment conditions were determined by semi-quantitative RT-PCR analyses. For each gene, the upper seven amplification bands represent amplified products from leaves of ‘Kyoho’ after treatment with 100 μM ABA; the bands under them represent amplified products from control leaves. B. Expression patterns of three randomly selected AP genes were determined by real-time PCR. *VvCEF4* was used as a positive control. Click here for file

Additional file 11 The cellular localization of grape APs. Click here for file

Additional file 12 **Comparison of expression pattern of orthologous AP pairs under various stresses and treatments.** Available expression patterns of *AtAP* genes based on microarray analysis and that of *VvAP* genes generated by semi-quantitative RT-PCR were compared. Click here for file

## References

[B1] Barrett AJ Cellular proteolysis - an overview Ann NY Acad Sci 1992 674 1 15 10.1111/j.1749-6632.1992.tb27472.x 1288356

[B2] Davies DR The structure and function of the aspartic proteinases Annu Rev Biophys Biophys Chem 1990 19 189 215 10.1146/annurev.bb.19.060190.001201 2194475

[B3] Rawlings ND Barrett AJ MEROPS: the peptidase database Nucleic Acids Res 1999 27 1 325 331 10.1093/nar/27.1.325 9847218 PMC148173

[B4] John K Kostka V Aspartic proteinases and their inhibitors FEBS Advanced Course 1985 Berlin: Walter de Gruyter 1 17

[B5] Flotman B Kostka V Comments on the nomenclature of aspartic proteinases FEBS Advanced Course 1985 Berlin: Walter de Gruyter 19 26

[B6] Simoes I Faro C Structure and function of plant aspartic proteinases Eur J Biochem 2004 271 11 2067 2075 10.1111/j.1432-1033.2004.04136.x 15153096

[B7] Faro C Gal S Aspartic proteinase content of the *Arabidopsis* genome Curr Protein Pept Sc 2005 6 6 493 500 10.2174/138920305774933268 16381599

[B8] Mutlu A Gal S Plant aspartic proteinases: enzymes on the way to a function Physiol Plantarum 1999 105 3 569 576 10.1034/j.1399-3054.1999.105324.x

[B9] Chen FQ Foolad MR Molecular organization of a gene in barley which encodes a protein similar to aspartic protease and its specific expression in nucellar cells during degeneration Plant Mol Biol 1997 35 6 821 831 10.1023/A:1005833207707 9426602

[B10] Prasad BD Creissen G Lamb C Chattoo BB Heterologous expression and characterization of recombinant OsCDR1, a rice aspartic proteinase involved in disease resistance Protein Expres Purif 2010 72 2 169 174 10.1016/j.pep.2010.03.018 20347986

[B11] Xia YJ Suzuki H Borevitz J Blount J Guo ZJ Patel K Dixon RA Lamb C An extracellular aspartic protease functions in *Arabidopsis* disease resistance signaling EMBO J 2004 23 4 980 988 10.1038/sj.emboj.7600086 14765119 PMC380998

[B12] Jaillon O Aury JM Noel B Policriti A Clepet C Casagrande A Choisne N Aubourg S Vitulo N Jubin C The grapevine genome sequence suggests ancestral hexaploidization in major angiosperm phyla Nature 2007 449 7161 463 465 10.1038/nature06148 17721507

[B13] Velasco R Zharkikh A Troggio M Cartwright DA Cestaro A Pruss D Pindo M FitzGerald LM Vezzulli S Reid J A high quality draft consensus sequence of the genome of a heterozygous grapevine variety Plos One 2007 2 12 e1326 10.1371/journal.pone.0001326 18094749 PMC2147077

[B14] Goff SA Ricke D Lan TH Presting G Wang RL Dunn M Glazebrook J Sessions A Oeller P Varma H A draft sequence of the rice genome (*Oryza sativa* L. ssp japonica) Science 2002 296 5565 92 100 10.1126/science.1068275 11935018

[B15] Tuskan GA DiFazio S Jansson S Bohlmann J Grigoriev I Hellsten U Putnam N Ralph S Rombauts S Salamov A The genome of black cottonwood, *Populus trichocarpa* (Torr. & Gray) Science 2006 313 5793 1596 1604 10.1126/science.1128691 16973872

[B16] Riechmann JL Heard J Martin G Reuber L Jiang CZ Keddie J Adam L Pineda O Ratcliffe OJ Samaha RR *Arabidopsis* transcription factors: genome-wide comparative analysis among eukaryotes Science 2000 290 5499 2105 2110 10.1126/science.290.5499.2105 11118137

[B17] Zhang YC Mao LY Wang H Brocker C Yin XJ Vasiliou V Fei ZJ Wang XP Genome-wide identification and analysis of grape aldehyde dehydrogenase (ALDH) gene superfamily Plos One 2012 7 2 e32153 10.1371/journal.pone.0032153 22355416 PMC3280228

[B18] Larkin MA Blackshields G Brown NP Chenna R McGettigan PA McWilliam H Valentin F Wallace IM Wilm A Lopez R Clustal W and Clustal X version 2.0 Bioinformatics 2007 23 21 2947 2948 10.1093/bioinformatics/btm404 17846036

[B19] Ge XC Dietrich C Matsuno M Li GJ Berg H Xia YJ An *Arabidopsis* aspartic protease functions as an anti-cell-death component in reproduction and embryogenesis EMBO Rep 2005 6 3 282 288 10.1038/sj.embor.7400357 15723040 PMC1299267

[B20] Yao X Xiong W Ye TT Wu Y Overexpression of the aspartic protease ASPG1 gene confers drought avoidance in *Arabidopsis* J Exp Bot 2012 63 7 2579 2593 10.1093/jxb/err433 22268147 PMC3346222

[B21] Tamura K Peterson D Peterson N Stecher G Nei M Kumar S MEGA5: molecular evolutionary genetics analysis using maximum likelihood, evolutionary distance, and maximum parsimony methods Mol Biol Evol 2011 28 10 2731 2739 10.1093/molbev/msr121 21546353 PMC3203626

[B22] Letunic I Doerks T Bork P SMART 7: recent updates to the protein domain annotation resource Nucleic Acids Res 2012 40 D1 D302 D305 10.1093/nar/gkr931 22053084 PMC3245027

[B23] Ren J Wen LP Gao XJ Jin CJ Xue Y Yao XB DOG 1.0: illustrator of protein domain structures Cell Res 2009 19 2 271 273 10.1038/cr.2009.6 19153597

[B24] Rice P Longden I Bleasby A EMBOSS: the European molecular biology open software suite Trends Genet 2000 16 6 276 277 10.1016/S0168-9525(00)02024-2 10827456

[B25] Guo AY Zhu QH Chen X Luo JC GSDS: a gene structure display server Yi Chuan 2007 29 8 1023 1026 10.1360/yc-007-1023 17681935

[B26] Boneh U Biton I Zheng CL Schwartz A Ben-Ari G Characterization of potential ABA receptors in *Vitis vinifera* Plant Cell Rep 2012 31 2 311 321 10.1007/s00299-011-1166-z 22016084

[B27] Upreti KK Murti GSR Response of grape rootstocks to salinity: changes in root growth, polyamines and abscisic acid Biol Plantarum 2010 54 4 730 734 10.1007/s10535-010-0130-z

[B28] Cramer GR Ergul A Grimplet J Tillett RL Tattersall EAR Bohlman MC Vincent D Sonderegger J Evans J Osborne C Water and salinity stress in grapevines: early and late changes in transcript and metabolite profiles Funct Integr Genomic 2007 7 2 111 134 10.1007/s10142-006-0039-y 17136344

[B29] Yang YZ He MY Zhu ZG Li SX Xu Y Zhang CH Singer SD Wang YJ Identification of the dehydrin gene family from grapevine species and analysis of their responsiveness to various forms of abiotic and biotic stress BMC Plant Biol 2012 12 140 10.1186/1471-2229-12-140 22882870 PMC3460772

[B30] Li HE Xu Y Xiao Y Zhu ZG Xie XQ Zhao HQ Wang YJ Expression and functional analysis of two genes encoding transcription factors, VpWRKY1 and VpWRKY2, isolated from Chinese wild *Vitis pseudoreticulata* Planta 2010 232 6 1325 1337 10.1007/s00425-010-1258-y 20811906

[B31] Wang LJ Li SH Thermotolerance and related antioxidant enzyme activities induced by heat acclimation and salicylic acid in grape (*Vitis vinifera* L.) leaves Plant Growth Regul 2006 48 2 137 144 10.1007/s10725-005-6146-2

[B32] Xiao HG Nassuth A Stress- and development-induced expression of spliced and unspliced transcripts from two highly similar dehydrin 1 genes in V, riparia and V, vinifera Plant Cell Rep 2006 25 9 968 977 10.1007/s00299-006-0151-4 16552595

[B33] Wang Y Liu Y He P Chen J Lamikanra O Lu J Evaluation of foliar resistance to *uncinula necator* in Chinese wild *Vitis* species Vitis 1995 34 3 159 164

[B34] Volker HDC Jose LR Omaira P Michael FT James ZZ Transcription factor CBF4 is a regulator of drought adaptation in *Arabidopsis* Plant Physiol 2002 130 639 648 10.1104/pp.006478 12376631 PMC166593

[B35] Mahbuba SAN *Vitis* CBF1 and *Vitis* CBF4 differ in their effect on *Arabidopsis* abiotic stress tolerance, development and gene expression Plant Cell Environ 2011 34 1345 1359 10.1111/j.1365-3040.2011.02334.x 21486303

[B36] Mohsen HLD Romain F Samia D Celine L Francois B Abdelwahed G Ahmed M Said H Identification and characterization of ‘rd22’ dehydration responsive gene in grapevine (*Vitis vinifera* L.) C R Biol 2008 331 8 569 578 10.1016/j.crvi.2008.05.002 18606386

[B37] Wang Q Zhang YC Gao M Jiao C Wang XP Identification and expression analysis of a pathogen-responsive PR-1 gene from Chinese wild *Vitis quinquangularis* Afr J Biotechnol 2011 10 75 17062 17069

[B38] Loake G Plant cell death: unmasking the gatekeepers Curr Biol 2001 11 24 R1028 R1031 10.1016/S0960-9822(01)00617-0 11747841

[B39] Brodersen P Petersen M Nielsen HB Zhu SJ Newman MA Shokat KM Rietz S Parker J Mundy J *Arabidopsis* MAP kinase 4 regulates salicylic acid- and jasmonic acid/ethylene-dependent responses via EDS1 and PAD4 Plant J 2006 47 4 532 546 10.1111/j.1365-313X.2006.02806.x 16813576

[B40] Chong JL Le Henanff G Bertsch C Walter B Identification, expression analysis and characterization of defense and signaling genes in *Vitis vinifera* Plant Physiol Bioch 2008 46 4 469 481 10.1016/j.plaphy.2007.09.010 17988883

[B41] Zhang JJ Wang YJ Wang XP Yang KQ Yang JX An improved method for rapidly extracting total RNA from *Vitis* Fruit Sci 2003 53 771 787

[B42] Kapushesky M Emam I Holloway E Kurnosov P Zorin A Malone J Rustici G Williams E Parkinson H Brazma A Gene expression atlas at the European bioinformatics institute Nucleic Acids Res 2010 38 D690 D698 10.1093/nar/gkp936 19906730 PMC2808905

[B43] Schmid M Davison TS Henz SR Pape UJ Demar M Vingron M Scholkopf B Weigel D Lohmann JU A gene expression map of *Arabidopsis thaliana* development Nature Genet 2005 37 5 501 506 10.1038/ng1543 15806101

[B44] Kenta NMK A knowledge base for predicting protein localization sites in eukaryotic cells Genomics 1992 14 4 897 911 10.1016/S0888-7543(05)80111-9 1478671 PMC7134799

[B45] Cannon SB Mitra A Baumgarten A Yong ND May G The roles of segmental and tandem gene duplication in the evolution of large gene families in *Arabidopsis thaliana* BMC Plant Biol 2004 4 10 10.1186/1471-2229-4-10 15171794 PMC446195

[B46] Chen JJ Ouyang YD Wang L Xie WB Zhang QF Aspartic proteases gene family in rice: gene structure and expression, predicted protein features and phylogenetic relation Gene 2009 442 1–2 108 118 19409457 10.1016/j.gene.2009.04.021

[B47] Barrett AJ Rawlings ND Woessner JF Handbook of proteolytic enzymes 2004 Amsterdam: Elsevier Academic Press

[B48] Munoz FF Mendieta JR Pagano MR Paggi RA Daleo GR Guevara MG The swaposin-like domain of potato aspartic protease (StAsp-PSI) exerts antimicrobial activity on plant and human pathogens Peptides 2010 31 5 777 785 10.1016/j.peptides.2010.02.001 20153392

[B49] Bari R Jones J Role of plant hormones in plant defence responses Plant Mol Biol 2009 69 4 473 488 10.1007/s11103-008-9435-0 19083153

[B50] Finkelstein RR Gampala SSL Rock CD Abscisic acid signaling in seeds and seedlings Plant Cell 2002 14 S15 S45 12045268 10.1105/tpc.010441 PMC151246

[B51] Xu GX Guo CC Shan HY Kong HZ Divergence of duplicate genes in exon-intron structure P Natl Acad Sci USA 2012 109 4 1187 1192 10.1073/pnas.1109047109 PMC3268293 22232673

[B52] Doyle JJ Flagel LE Paterson AH Rapp RA Soltis DE Soltis PS Wendel JF Evolutionary genetics of genome merger and doubling in plants Annu Rev Genet 2008 42 443 461 10.1146/annurev.genet.42.110807.091524 18983261

[B53] Gambetta GA Matthews MA Shaghasi TH McElrone AJ Castellarin SD Sugar and abscisic acid signaling orthologs are activated at the onset of ripening in grape Planta 2010 232 1 219 234 10.1007/s00425-010-1165-2 20407788 PMC2872022

[B54] Hurley I Hale ME Prince VE Duplication events and the evolution of segmental identity Evol Dev 2005 7 6 556 567 10.1111/j.1525-142X.2005.05059.x 16336409

[B55] Kellis M Birren BW Lander ES Proof and evolutionary analysis of ancient genome duplication in the yeast *Saccharomyces cerevisiae* Nature 2004 428 6983 617 624 10.1038/nature02424 15004568

[B56] Wolfe KH Shields DC Molecular evidence for an ancient duplication of the entire yeast genome Nature 1997 387 6634 708 713 10.1038/42711 9192896

[B57] Li WY Liu B Yu LJ Feng DR Wang HB Wang JF Phylogenetic analysis, structural evolution and functional divergence of the 12-oxo-phytodienoate acid reductase gene family in plants BMC Evol Biol 2009 9 90 10.1186/1471-2148-9-90 19416520 PMC2688005

[B58] Wapinski I Pfeffer A Friedman N Regev A Natural history and evolutionary principles of gene duplication in fungi Nature 2007 449 7158 54 U36 10.1038/nature06107 17805289

[B59] Smith AD Sumazin P Xuan ZY Zhang MQ DNA motifs in human and mouse proximal promoters predict tissue-specific expression P Natl Acad Sci USA 2006 103 16 6275 6280 10.1073/pnas.0508169103 PMC1458868 16606849

[B60] Wang DY Sung HM Wang TY Huang CJ Yang P Chang T Wang YC Tseng DL Wu JP Lee TC Expression evolution in yeast genes of single-input modules is mainly due to changes in transacting factors Genome Res 2007 17 8 1161 1169 10.1101/gr.6328907 17615293 PMC1933509

[B61] Xing Y Ouyang ZQ Kapur K Scott MP Wong WH Assessing the conservation of mammalian gene expression using high-density exon arrays Mol Biol Evol 2007 24 6 1283 1285 10.1093/molbev/msm061 17387099

[B62] Chen ZJ Ni ZF Mechanisms of genomic rearrangements and gene expression changes in plant polyploids Bioessays 2006 28 3 240 252 10.1002/bies.20374 16479580 PMC1986666

[B63] Rapp RA Wendel JF Epigenetics and plant evolution New Phytol 2005 168 1 81 91 10.1111/j.1469-8137.2005.01491.x 16159323

[B64] Zhang XY Yazaki J Sundaresan A Cokus S Chan SWL Chen HM Henderson IR Shinn P Pellegrini M Jacobsen SE Genome-wide high-resolution mapping and functional analysis of DNA methylation in *Arabidopsis* Cell 2006 126 6 1189 1201 10.1016/j.cell.2006.08.003 16949657

[B65] Zilberman D Gehring M Tran RK Ballinger T Henikoff S Genome-wide analysis of *Arabidopsis thaliana* DNA methylation uncovers an interdependence between methylation and transcription Nature Genet 2007 39 1 61 69 10.1038/ng1929 17128275

[B66] Blanc G Wolfe KH Functional divergence of duplicated genes formed by polyploidy during *Arabidopsis* evolution Plant Cell 2004 16 7 1679 1691 10.1105/tpc.021410 15208398 PMC514153

[B67] Zhang JZ Evolution by gene duplication: an update Trends Ecol Evol 2003 18 6 292 298 10.1016/S0169-5347(03)00033-8

[B68] Dunn BM Structure and mechanism of the pepsin-like family of aspartic peptidases Chem Rev 2002 102 12 4431 4458 10.1021/cr010167q 12475196

[B69] Timotijevic GS Milisavljevic MD Radovic SR Konstantinovic MM Maksimovic VR Ubiquitous aspartic proteinase as an actor in the stress response in buckwheat J Plant Physiol 2010 167 1 61 68 10.1016/j.jplph.2009.06.017 19643510

[B70] Wang YP Wang XY Tang HB Tan X Ficklin SP Feltus FA Paterson AH Modes of gene duplication contribute differently to genetic novelty and redundancy, but show parallels across divergent angiosperms Plos One 2011 6 12 e28150 10.1371/journal.pone.0028150 22164235 PMC3229532

[B71] Lyons E Pedersen B Kane J Alam M Ming R Tang HB Wang XY Bowers J Paterson A Lisch D Freeling M Finding and comparing syntenic regions among Arabidopsis and the outgroups papaya, poplar, and grape: CoGe with rosids Plant Physiol 2008 148 1772 1781 10.1104/pp.108.124867 18952863 PMC2593677

[B72] Zhang YC Gao M Singer SD Fei ZJ Wang H Wang XP Genome-wide identification and analysis of the tify gene family in grape Plos One 2012 7 9 e44465 10.1371/journal.pone.0044465 22984514 PMC3439424

[B73] Ling J Jiang WJ Zhang Y Yu HJ Mao ZC Gu XF Huang SW Xie BY Genome-wide analysis of WRKY gene family in *Cucumis sativus* BMC Genomics 2011 12 471 10.1186/1471-2164-12-471 21955985 PMC3191544

[B74] Timotijevic GS Milisavljevic MD Radovic SR Konstantinovic MM Maksimovic VR Seed-specific aspartic proteinase feap12 from buckwheat (*Fagopyrum esculentum* Moench) Arch Biol Sci 2010 62 1 143 151 10.2298/ABS1001143T

[B75] de Carvalho MHC d’Arcy-Lameta A Roy-Macauley H Gareil M El Maarouf H Pham-Thi AT Zuily-Fodil Y Aspartic protease in leaves of common bean (*Phaseolus vulgaris* L.) and cowpea (*Vigna unguiculata* L. Walp): enzymatic activity, gene expression and relation to drought susceptibility FEBS Lett 2001 492 3 242 246 10.1016/S0014-5793(01)02259-1 11257502

[B76] Rodrigo I Vera P Conejero V Degradation of tomato pathogenesis-related proteins by an endogenous 37-kDa aspartyl proteinase Eur J Biochem 1989 184 663 669 10.1111/j.1432-1033.1989.tb15064.x 2680484

[B77] Rodrigo I Vera P Vanloon LC Conejero V Degradation of tobacco pathogenesis-related proteins – evidence for conserved mechanisms of degradation of pathogenesis-related proteins in plants Plant Physiol 1991 95 616 622 10.1104/pp.95.2.616 16668027 PMC1077576

[B78] Guevara MG Oliva CR Huarte M Daleo GR An aspartic protease with antimicrobial activity is induced after infection and wounding in intercellular fluids of potato tubers Eur J Plant Pathol 2002 108 2 131 137 10.1023/A:1015049629736

[B79] Fung RWM Gonzalo M Fekete C Kovacs LG He Y Marsh E McIntyre LM Schachtman DP Qiu WP Powdery mildew induces defense-oriented reprogramming of the transcriptome in a susceptible but not in a resistant grapevine Plant Physiol 2008 146 1 236 249 17993546 10.1104/pp.107.108712 PMC2230561

[B80] Beers EP Programmed cell death during plant growth and development Cell Death Differ 1997 4 8 649 661 10.1038/sj.cdd.4400297 16465277

[B81] Terauchi K Asakura T Nishizawa NK Matsumoto I Abe K Characterization of the genes for two soybean aspartic proteinases and analysis of their different tissue-dependent expression Planta 2004 218 6 947 957 10.1007/s00425-003-1179-0 14727111

[B82] Runebergroos P Kervinen J Kovaleva V Raikhel NV Gal S The aspartic proteinase of barley is a vacuolar enzyme that processes probarley lectin in-vitro Plant Physiol 1994 105 1 321 329 10.1104/pp.105.1.321 8029356 PMC159360

[B83] Ramalho-Santos M Verissimo P Cortes L Samyn B Van Beeumen J Pires E Faro C Identification and proteolytic processing of procardosin A Eur J Biochem 1998 255 1 133 138 10.1046/j.1432-1327.1998.2550133.x 9692911

[B84] Schaaf A Reski R Decker EL A novel aspartic proteinase is targeted to the secretory pathway and to the vacuole in the moss Physcomitrella patens Eur J Cell Biol 2004 83 4 145 152 10.1078/0171-9335-00371 15260436

[B85] Pereira CS da Costa DS Pereira S Nogueira FD Albuquerque PM Teixeira J Faro C Pissarra J Cardosins in postembryonic development of cardoon: towards an elucidation of the biological function of plant aspartic proteinases Protoplasma 2008 232 3–4 203 213 18767217 10.1007/s00709-008-0288-9

[B86] Vieira M Pissarra J Verissimo P Castanheira P Costa Y Pires E Faro C Molecular cloning and characterization of cDNA encoding cardosin B, an aspartic proteinase accumulating extracellularly in the transmitting tissue of *Cynara cardunculus* L Plant Mol Biol 2001 45 5 529 539 10.1023/A:1010675015318 11414612

[B87] da Costa DS Pereira S Moore I Pissarra J Dissecting cardosin B trafficking pathways in heterologous systems Planta 2010 232 6 1517 1530 10.1007/s00425-010-1276-9 20872011

[B88] Kato Y Murakami S Yamamoto Y Chatani H Kondo Y Nakano T Yokota A Sato F The DNA-binding protease, CND41, and the degradation of ribulose-1,5-bisphosphate carboxylase/oxygenase in senescent leaves of tobacco Planta 2004 220 1 97 104 10.1007/s00425-004-1328-0 15252735

[B89] Murakami S Kondo Y Nakano T Sato F Protease activity of CND41, a chloroplast nucleoid DNA-binding protein, isolated from cultured tobacco cells FEBS Lett 2000 468 1 15 18 10.1016/S0014-5793(00)01186-8 10683432

[B90] Nakano T Sato F Yamada Y Analysis of nucleoid-proteins in tobacco chloroplasts Plant Cell Physiol 1993 34 6 873 880

[B91] Diaz C Lemaitre T Christ A Azzopardi M Kato Y Sato F Morot-Gaudry JF Le Dily F Masclaux-Daubresse C Nitrogen recycling and remobilization are differentially controlled by leaf senescence and development stage in *Arabidopsis* under low nitrogen nutrition Plant Physiol 2008 147 3 1437 1449 10.1104/pp.108.119040 18467460 PMC2442554

[B92] Kato Y Yamamoto Y Murakami S Sato F Post-translational regulation of CND41 protease activity in senescent tobacco leaves Planta 2005 222 4 643 651 10.1007/s00425-005-0011-4 16021504

[B93] Kato YSN Yamamoto Y Sato F van der Est A, Bruce D, Lawrence BD Regulation of senescence by aspartic protease: CND41 in tobacco and CND41 homologues in *Arabidopsis* Photosynthesis: Fundamental Aspects to Global Perspectives: Proceedings of the 13th International Congress on Photosynthesis 2005 KS: Alliance Communications Group 821 823

[B94] Paparelli E Gonzali S Parlanti S Novi G Giorgi FM Licausi F Kosmacz M Feil R Lunn JE Brust H Misexpression of a chloroplast aspartyl protease leads to severe growth defects and alters carbohydrate metabolism in *Arabidopsis* Plant Physiol 2012 160 3 1237 1250 10.1104/pp.112.204016 22987884 PMC3490589

[B95] Almeida CM Pereira C da Costa DS Pereira S Pissarra J Simoes I Faro C Chlapsin, a chloroplastidial aspartic proteinase from the green algae *Chlamydomonas reinhardtii* Planta 2012 236 1 283 296 10.1007/s00425-012-1605-2 22349731

