# Supplementary figures and images for: Genome-wide identification, evolutionary and expression analysis of the aspartic protease gene superfamily in grape
Source: BMC Genomics. 2013 Aug 15;14:554. doi: 10.1186/1471-2164-14-554 (PMC3751884; doi:10.1186/1471-2164-14-554)

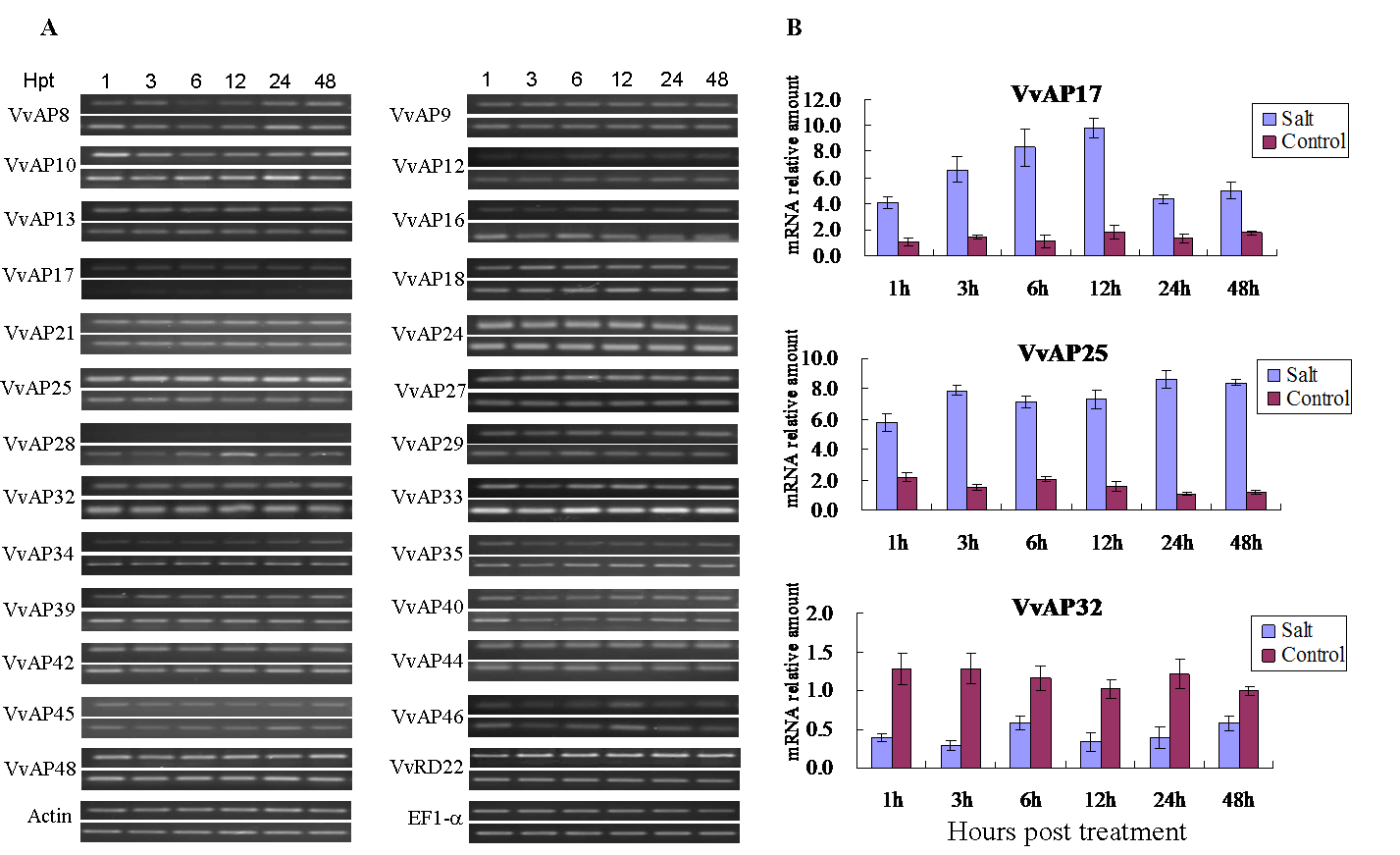

Supplement: Additional file 6 — Expression patterns of grape AP genes under salt treatment condition. A. Expression patterns of 25 AP genes under salt treatment conditions were determined by semi-quantitative RT-PCR analyses. For each gene, the upper six amplification bands represent amplified products from leaves of ‘Kyoho’ after treatment with 2 dm3 250 mM NaCl; the bands under them represent amplified products from leaves of the control. B. Expression patterns of three randomly selected AP genes were analyzed by real-time RT-PCR. The grape Actin1 and EF1-α genes were used as internal controls to normalize the data. VvRD22 served as a positive control for salt stress. The error bars were calculated based on three replicates. [file 1471-2164-14-554-S6.png]

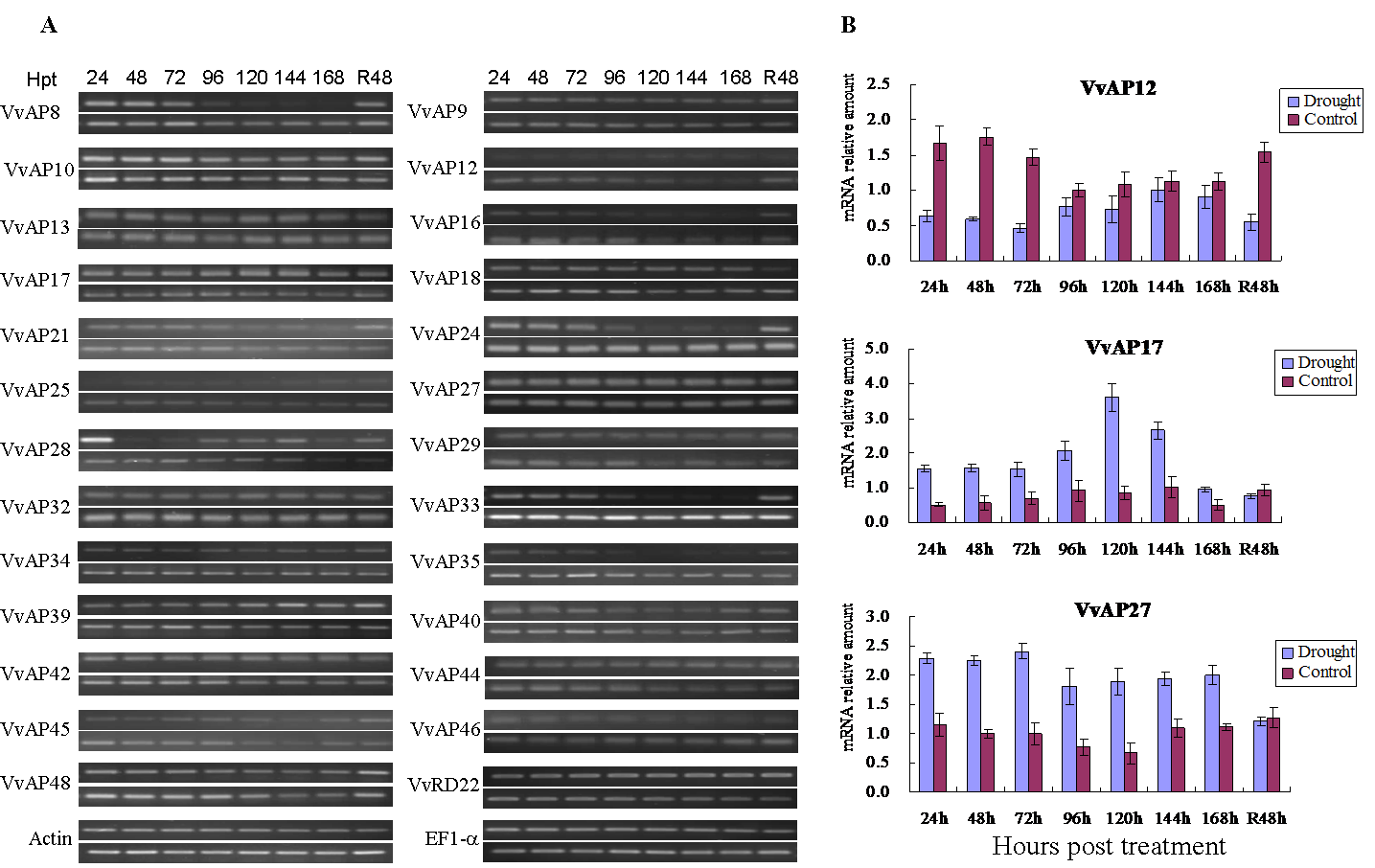

Supplement: Additional file 7 — Expression patterns of grape AP genes under drought treatment. A. Expression patterns of 25 AP genes under drought treatment conditions were determined by semi-quantitative RT-PCR analyses. For each gene, the upper eight amplification bands represent amplified products from leaves of ‘Kyoho’ under drought stress for 24 h, 48 h, 72 h, 96 h, 120 h, 144 h, 168 h after 48 h of recovery (R48; rewatered); the bands under them represent amplified products from leaves of the control. B. Expression patterns of three randomly selected AP genes were detected by real-time PCR. VvRD22 was used as a positive control. [file 1471-2164-14-554-S7.png]

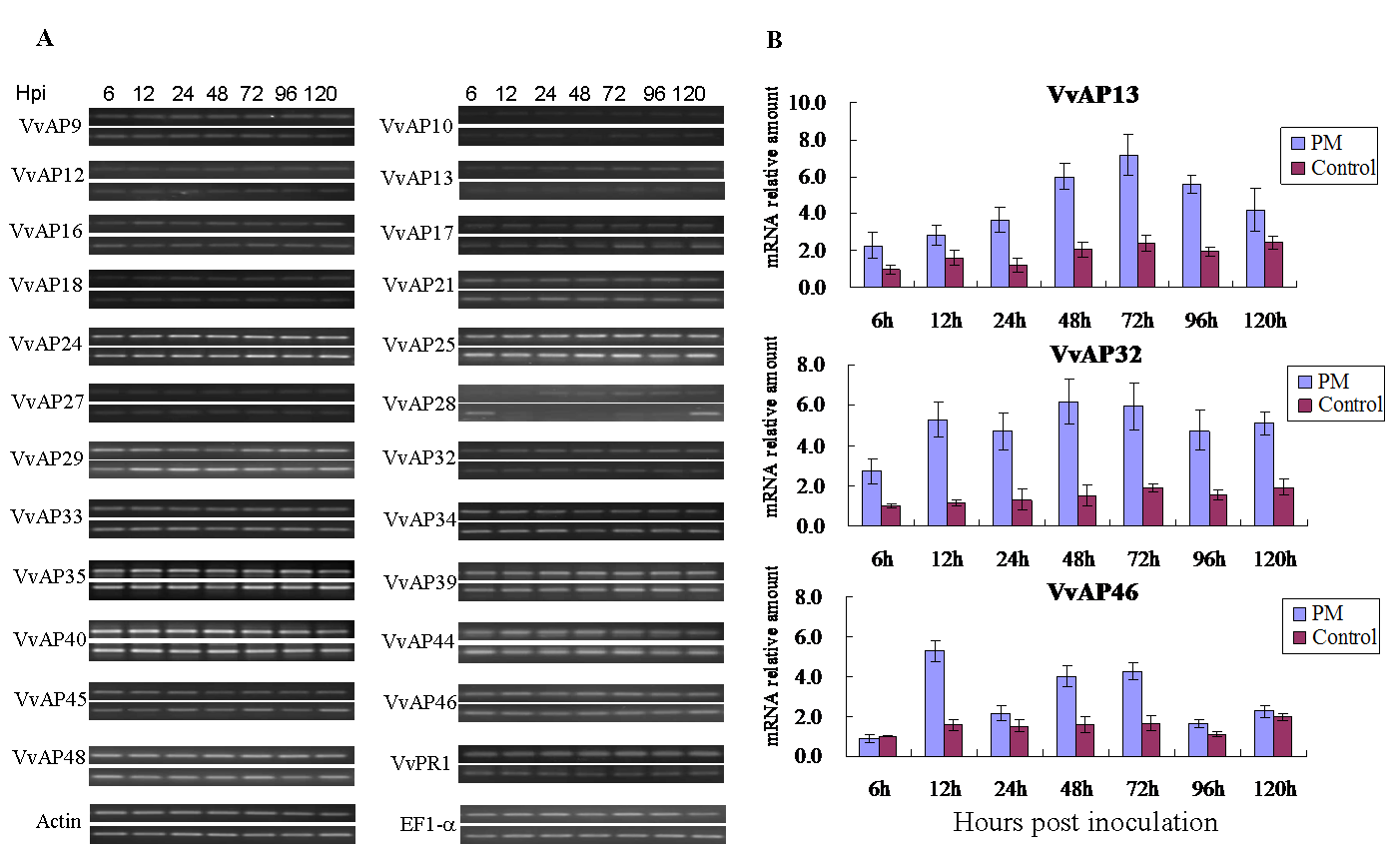

Supplement: Additional file 8 — Expression patterns of grape AP genes under powdery mildew treatment. A. Expression patterns of 23 AP genes under PM treatment condition were determined by semi-quantitative RT-PCR analyses. For each gene, the upper seven amplification bands represent amplified products from leaves of ‘Shang-24’ after inocululation with powdery mildew; the bands under them represent amplified products from Mock-inoculated leaves. B. Expression patterns of three randomly selected AP genes were detected by real-time PCR. VvPR1 was used as a positive control. [file 1471-2164-14-554-S8.png]

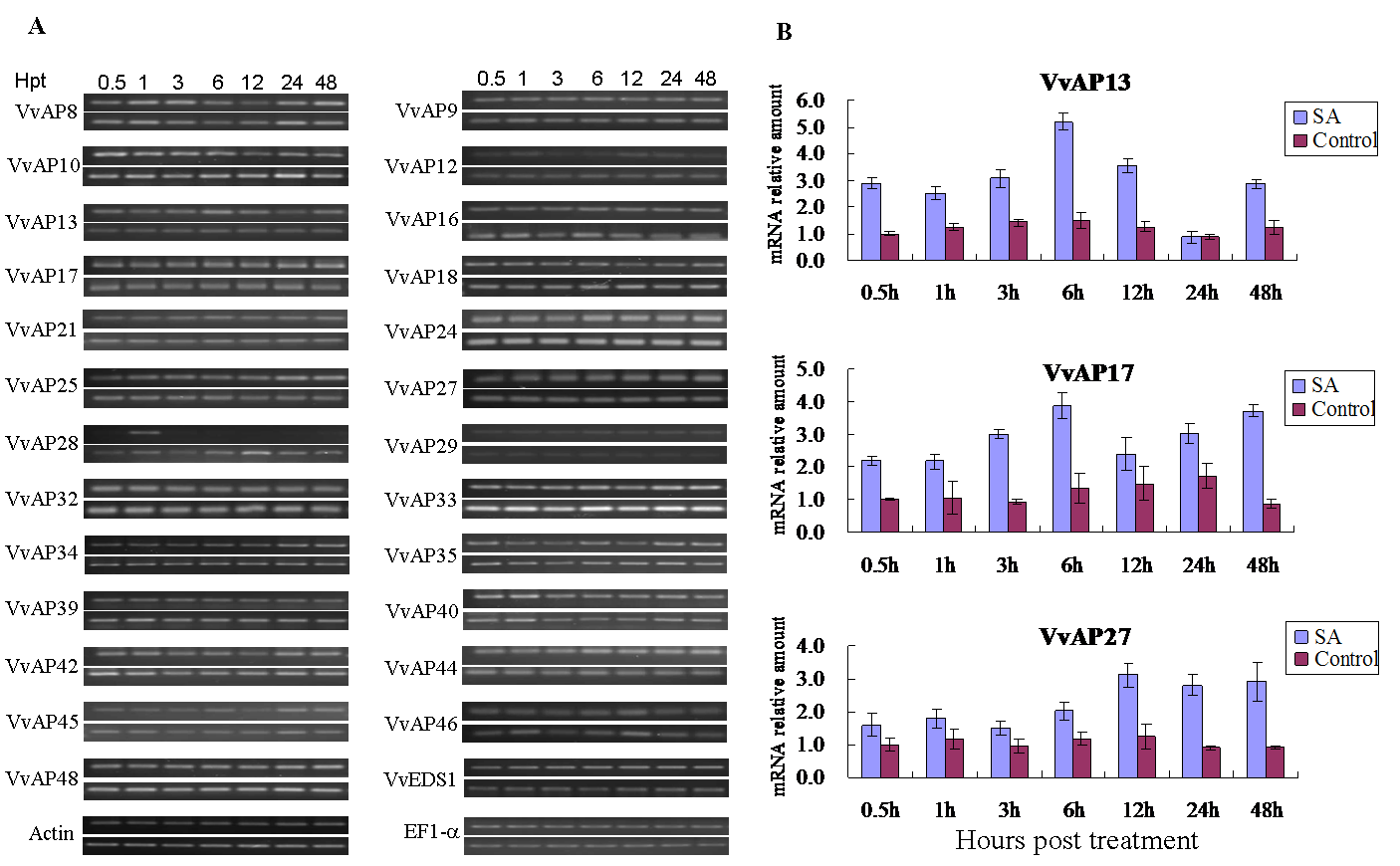

Supplement: Additional file 9 — Expression patterns of grape AP genes under SA treatment. A. Expression patterns of 25 AP genes under SA treatment conditions were determined by semi-quantitative RT-PCR analyses. For each gene, the upper seven amplification bands represent amplified products from leaves of ‘Kyoho’ after treatment with 100 μM SA; the bands under them represent amplified products from control leaves. B. Expression patterns of three randomly selected AP genes were analyzed by real-time PCR. VvEDS1 was used as a positive control. [file 1471-2164-14-554-S9.png]

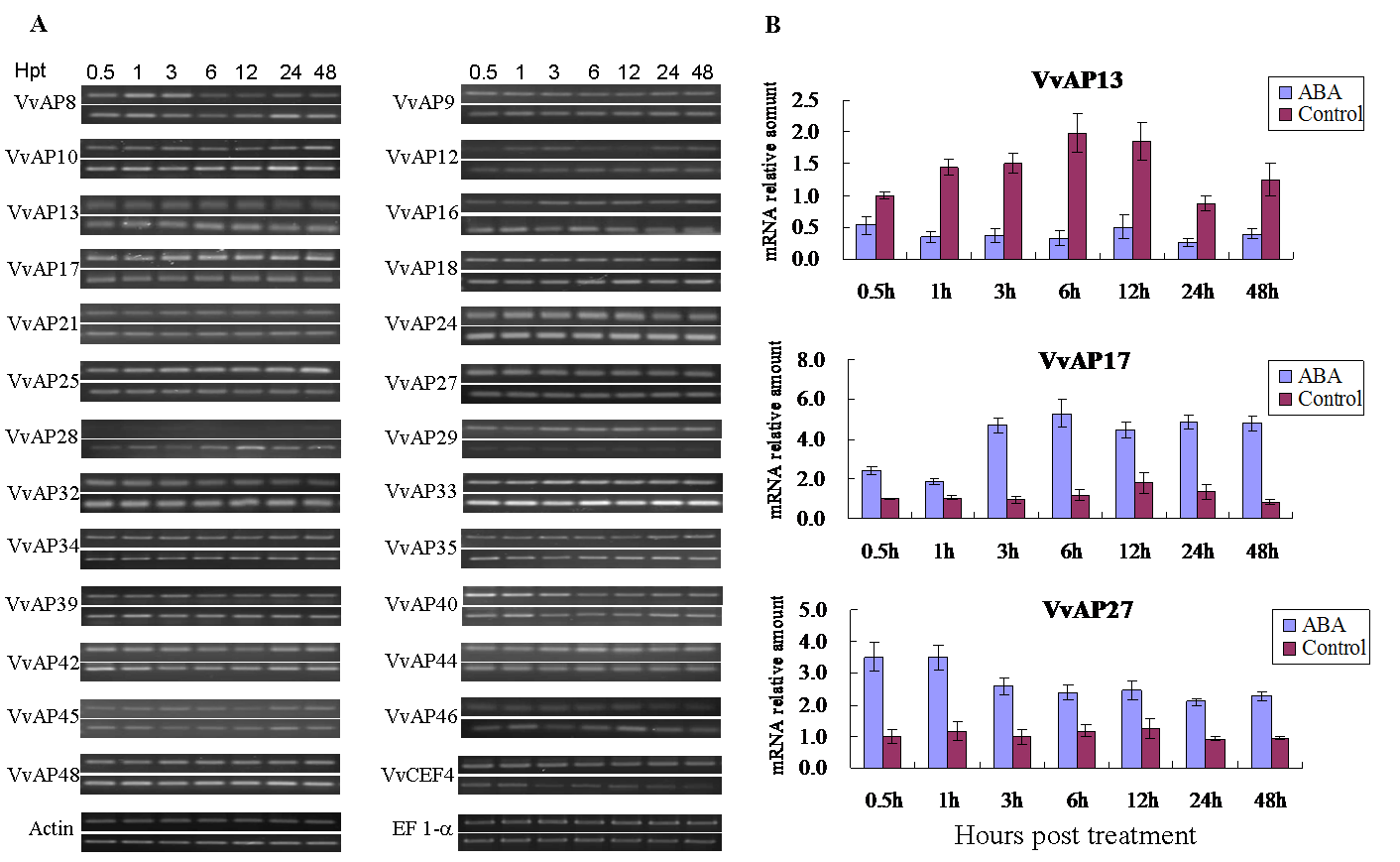

Supplement: Additional file 10 — Expression patterns of grape AP genes under ABA treatment. A. Expression patterns of 25 AP genes under ABA treatment conditions were determined by semi-quantitative RT-PCR analyses. For each gene, the upper seven amplification bands represent amplified products from leaves of ‘Kyoho’ after treatment with 100 μM ABA; the bands under them represent amplified products from control leaves. B. Expression patterns of three randomly selected AP genes were determined by real-time PCR. VvCEF4 was used as a positive control. [file 1471-2164-14-554-S10.png]
